# Imputation of missing covariate in randomized controlled trials with a continuous outcome: Scoping review and new results

**DOI:** 10.1002/pst.2041

**Published:** 2020-06-08

**Authors:** Mutamba T. Kayembe, Shahab Jolani, Frans E. S. Tan, Gerard J. P. van Breukelen

**Affiliations:** ^1^ Department of Methodology and Statistics, School CAPHRI, Care and Public Health Research Institute Maastricht University Maastricht the Netherlands

**Keywords:** mean imputation, missing covariate, missing‐indicator method, multiple imputation, randomized studies, review

## Abstract

In this article, we first review the literature on dealing with missing values on a covariate in randomized studies and summarize what has been done and what is lacking to date. We then investigate the situation with a continuous outcome and a missing binary covariate in more details through simulations, comparing the performance of multiple imputation (MI) with various simple alternative methods. This is finally extended to the case of time‐to‐event outcome. The simulations consider five different missingness scenarios: missing completely at random (MCAR), at random (MAR) with missingness depending only on the treatment, and missing not at random (MNAR) with missingness depending on the covariate itself (MNAR1), missingness depending on both the treatment and covariate (MNAR2), and missingness depending on the treatment, covariate and their interaction (MNAR3). Here, we distinguish two different cases: (1) when the covariate is measured before randomization (best practice), where only MCAR and MNAR1 are plausible, and (2) when it is measured after randomization but before treatment (which sometimes occurs in nonpharmaceutical research), where the other three missingness mechanisms can also occur. The proposed methods are compared based on the treatment effect estimate and its standard error. The simulation results suggest that the patterns of results are very similar for all missingness scenarios in case (1) and also in case (2) except for MNAR3. Furthermore, in each scenario for continuous outcome, there is at least one simple method that performs at least as well as MI, while for time‐to‐event outcome MI is best.

## 1 INTRODUCTION

Important prognostic factors, in epidemiological and clinical studies, are frequently included as covariates in the statistical model for analyzing the relationship between a treatment or exposure and some health outcomes. This is done either to improve the precision of the treatment effect estimate by reducing residual outcome variance (in clinical trials), or to adjust for confounding (in observational studies), or because of interest in the covariate effects themselves (all studies). This practice is commonly known as adjustment for covariates.^1^, ^2^, ^3^ However, it is often difficult to completely observe these covariates, especially in lengthy patient interviews. This results in occurrences of missing data in the covariates, which complicate the adjustment process.^4^, ^5^ Complete case analysis (CCA), which omits all incomplete cases (IC) from the analysis, has been shown to often produce biased and/or less powerful results, though White and Carlin^5^ reported unbiasedness of CCA under some specific scenarios in the context of regression analysis.

Missing covariate data raise different issues in randomized studies than in nonrandomized studies. In the former, unlike in the latter, the predictor of interest (treatment group) is independent of all baseline (prerandomization) covariates (both observed and unobserved). Consequently, optimal (or suboptimal) methods for handling missing covariate data in nonrandomized studies cannot necessarily be expected to be optimal (or suboptimal) in randomized studies.^4^ Indeed, as an example, the missing‐indicator method (described in section 2.2.3 of this study) has been shown to produce unbiased treatment effect estimates in randomized studies but can introduce bias in nonrandomized studies.^6^ We note that this finding was based on a scenario where the analysis of interest was linear regression, and missingness of the covariate was independent of treatment or any postrandomization variable.

To better understand the argument above, we need to distinguish the different missingness mechanisms that can occur. Data can be missing completely at random (MCAR), at random (MAR), or, not at random (MNAR). Data are MCAR if participants who have missing data are a random subset of the complete sample of participants, that is, the missingness is totally unrelated to any observed or unobserved participant data. Under MCAR, even a missing data method as simple as CCA produces unbiased results. Data are MAR if the missingness depends only on observed data and not on unobserved data given the observed data, for example, if missingness at a follow‐up is related to the health status of the patient at some preceding time point, or to a baseline covariate. In the latter case, MAR is sometimes called stratified or covariate‐dependent MCAR, depending on the author. In this article, we call it MAR. Under MAR, sophisticated methods such as multiple imputation (MI) and maximum likelihood (ML)‐based methods produce unbiased results under appropriate conditions.^7^, ^8^, ^9^, ^10^ Finally, data are MNAR if the missingness is (also) related to unobserved data, for example, if the value of a participant characteristic is missing because of that value itself, such as when patients in a trial drop out because of sudden worsening of their health just before the planned next measurement. Under MNAR, none of the aforementioned missing data methods can universally guarantee unbiased results.

Missingness types may have different implications in randomized than in nonrandomized studies. In the former, unlike in the latter, randomization ensures that the distribution of baseline covariates (both observed and unobserved) will be the same in both or all treatment groups, apart from chance differences. Indeed, this holds regardless of whether or not the covariates are complete or incomplete. Thus, in randomized studies, unlike in nonrandomized studies, patients with any combination of missing and observed baseline values must be equally likely in each treatment group, irrespective of the missingness mechanism if this does not depend on treatment group (as is often but not always the case).^11^ ^, p 37^ Consequently, for treatments comparison, missing data methods (whose performance depend on missingness types, as explained in the previous paragraph), may perform differently in randomized studies than in nonrandomized studies. Indeed, as an example, in a trial with linear regression as the analysis model of interest, CCA will certainly be unbiased (even if the variable predictive of missingness is not included in the analysis) if the trial is randomized, but not necessarily if it is not.^11^ ^, p 37^ Note that the argument in this paragraph, about the implications of missingness types on the performance of missing data methods in randomized studies, is true for baseline covariates measured before randomization, but not necessarily for postrandomization covariates.

Despite the preceding argument, most studies on how to appropriately handle missing covariate data have generally based their conclusions on nonrandomized studies, when suggesting that sophisticated methods such as MI and ML‐based methods^9^, ^12^, ^13^ generally produce better results than simpler methods such as CCA and the missing‐indicator method.^4^ In this study, we investigate the problem of missing covariates in randomized controlled trials (RCTs). Those interested in the topic of missing covariates in observational studies, which is beyond the scope of the present article, are referred to the literature.^5^, ^14^, ^15^ In Section 2, we conduct a scoping review to identify the missingness scenarios and missing data methods that have been explored to date in the literature and summarize the findings. In Section 3, we fill some gaps in the literature by performing a simulation study with new combinations of missingness scenarios and missing data methods in the case of a continuous outcome. This is briefly extended to the case of a time‐to‐event outcome in Section 4. We conclude with a discussion in Section 5.

## 2 SCOPING REVIEW

Scoping reviews are used to identify, retrieve, and summarize literature related to a particular (emerging) research topic in order to highlight research gaps and potential avenues for future studies on that topic.^16^, ^17^ Unlike systematic reviews, where quantitative analyses can be utilized to glean trends in literature, scoping reviews assess the qualitative content of literature through concept and thematic mapping.^18^, ^19^

### 2.1 Method

The method we applied to scope review the literature involved: (1) specifying the criteria upon which articles should be included in the review; (2) searching for relevant articles based on the inclusion criteria; (3) classifying the studies found to highlight their differences in contents; and (4) identifying gaps in the literature. These four steps are described below.

Inclusion criteria:

- RCTs, as opposed to nonrandomized and cluster randomized studies;
- Quantitative outcome (as opposed to categorical outcome);
- Single outcome (as opposed to multiple outcomes);
- Missingness in a covariate;
- Simulation study (as opposed to application study), to ensure that (a) the true parameter values, notably the treatment effect, are known, and (b) complete data are available for comparison with the results of missing data methods applied to the data after missingness has been created, and (c) sampling error can be assessed by having a large number of samples from the same distribution.

Search method:

- We used Google Scholar and Web of Science to search the relevant articles. Google Scholar is the best‐known scientific search engine of articles from various disciplines. Web of Science contains bibliographic data of articles from about 9500 scientific journals. We utilized these two search tools to guarantee the comprehensiveness of our search.
- The precise keywords we utilized for our search were: missing (in the title), imputation (in the content), randomized trials (in the content), and simulation (in the content);
- The period of search used was for all years, starting from 2017 backward;
- We screened the abstract and the methodology of the articles found, to retain only articles that satisfied the inclusion criteria.
- To ensure that our systematic search was conducted efficiently, and that we did not miss (possibly due to our keywords) any relevant article, we subsequently used a snowballing approach: we consulted the references of all the relevant articles found in our search, starting with the most recently published article, assuming that all the authors would have consulted and included the relevant publications in their references. Each relevant article found in the references was subsequently consulted following the same process, and the oldest relevant article that we could find was published in 1990.

Classification of studies found by:

- Covariate types, with as types: (i) baseline measurement of the outcome, (ii) other continuous covariate, and (iii) categorical covariate;
- Missingness types (MCAR, MAR, and MNAR) studied;
- Methods for handling missingness applied.

Identification of gaps in the literature:

- First, based on all the dimensions of the classification stage above, we determined all possible study scenarios (ie, scenarios that were investigated plus those that were not investigated), with a study scenario defined as the combination of the covariate type (eg, categorical), missingness type (eg, MCAR) and method to deal with missing data in the covariate (eg, CCA);
- Second, we identified as gaps the scenarios that were not investigated in the reviewed articles.

### 2.2 Results

Only four articles^4^, ^6^, ^20^, ^21^ were found to be relevant for this review based on the inclusion criteria. Table 1 gives more details of these studies. Clearly, there has not been intensive research on this review's topic of interest.

**TABLE 1:** Details of the relevant publications identified for the review, with the corresponding covariate types and missingness mechanisms

No	First author	Year	Analysis model						Missingness mechanism		
			Outcome	Treatment group (0/1 coded)	Baseline covariates			Treatment‐by‐covariate interaction	MCAR	MAR	MNAR
			*Y*_1_	*T*	*Y*_0_	*X*_0_	*Z*_0_	*T* * *Y*_0_ or *T* * *X*_0_			
P1	Schemper et al^20^	1990	✓	✓	×	×	✓	×	✓	✓	✓
P2	White et al^4^	2005	✓	✓	✓	×	×	×	✓	×	✓
P3	Groenwold et al^6^	2012	✓	✓	✓	×	×	×	✓	×	✓
P4	Sullivan et al^21^	2016	✓	✓	✓	×	×	×	✓	×	✓

*Note*: Articles, from the oldest (P1) to the most recent (P4) ones are in the first column; each article's first author is in the second column; each article's year of publication is in the third column; the rest of the columns show, for each article, the analysis model (with variable types) and the missingness mechanisms; *Y*_1_, continuous outcome (posttreatment measurement); *Y*_0_, continuous baseline covariate (pretreatment measurement of *Y*_1_); *X*_0_, continuous baseline covariate (not pretreatment measurement of *Y*_1_); *Z*_0_, binary baseline covariate. The ticks per row indicate the setting (variables and missingness mechanisms) tackled by the article in that row, and the crosses indicate the variables and missingness mechanisms not used in that setting.

Abbreviations: MAR, missing at random; MCAR, missing completely at random; MNAR, missing not at random.

#### 2.2.1 Covariate type

With respect to whether or not the covariate used was a premeasurement of the outcome (see Table 1), the covariate used in P1 was not a premeasurement of the outcome and was categorical, while the covariate in P2, P3, and P4 was a premeasurement of the outcome and continuous. All studies considered scenarios with only one covariate.

#### 2.2.2 Missingness types

For the missingness mechanisms, Table 1 shows that every article covered the MCAR and MNAR mechanisms. MCAR was defined as the missingness being totally independent of treatment, pre‐, and post‐randomization variables. MNAR was defined as the missingness being dependent only on the value of the covariate itself. In addition to MCAR and MNAR, P1 analyzed a condition with missingness of the covariate being treatment‐dependent; which is sometimes called stratified MCAR,^22^ but falls under MAR in this review. We note that this scenario is possible only if the covariate is measured postrandomization, which might have been the case but was not explicitly stated in P1. MAR in the sense of covariate missingness depending on other covariates does not apply here since all articles in Table 1 used a model with only one covariate on top of the treatment itself. P2 and P4 used two variants of MNAR which were called informative missingness 1 and 2 (see P2 and P4 for details).

We emphasize that the missingness of the covariate may, or may not, depend on treatment, and that dependence can be prevented by measuring the covariate prerandomization, which is the dominant practice in pharmaceutical trials. However, measuring the covariate prerandomization is unfortunately not always done in randomized trials in health research (eg, in cluster randomized trials,^23^, ^24^ and in mental and public health research^25^, ^26^, ^27^) where the covariate is measured postrandomization but before treatment. In such cases, we can have dependence of the missingness in covariate on treatment, even if the covariate itself is not related to treatment. As an example, gender as covariate can be measured postrandomization and still be independent of treatment, while its missingness may depend on treatment. This may happen, for instance, in an RCT of face‐to‐face vs online treatment of depression, where participants skip some items in the online questionnaire among others about their gender.

#### 2.2.3 Methods for handling missingness

Methods for handling missing data utilized in every reviewed article are summarized in Table 2. Unadjusted analysis (UA) omits the incomplete covariate from the analysis so that the treatment effect is not adjusted for that covariate. CCA removes all cases with a missing covariate value from the analysis. The probability imputation technique (PIT) is the mean imputation of a binary covariate per treatment group, that is, missing covariate values within a treatment group are replaced with the mean of the observed covariate values within that group.^20^

**TABLE 2:** Details of the relevant publications identified for the review, with the corresponding methods used to handle missing covariate

No	First author	Year	Methods used to handle missing covariate data														
			UA	CCA	PIT	Mean imputation				Missing indicator				MI		LMM	
						I	IT	WI	WIT	M	MT	WM	WMT	O	T	S	M
P1	Schemper et al^20^	1990	✓	✓	✓	×	×	×	×	×	×	×	×	×	×	×	×
P2	White et al^4^	2005	✓	✓	×	✓	✓	✓	×	✓	✓	×	×	×	×	✓	✓
P3	Groenwold et al^6^	2012	×	✓	×	×	×	×	×	✓	×	×	×	✓	×	×	×
P4	Sullivan et al^21^	2016	✓	✓	×	✓	×	✓	×	✓	✓	×	×	✓	✓	✓	×

*Note*: The ticks per row indicate all the methods tackled by the article in that row, and the crosses indicate the methods not tackled by that article.

Abbreviations: CCA, complete cases analysis; I, overall mean imputation; IT, mean imputation by treatment group; LMMM, likelihood‐based mixed model with missing‐indicator. LMMS, likelihood‐based mixed model without missing‐indicator; M, missing‐indicator overall; MIO, multiple imputation overall; MIT, multiple imputation by treatment group; MT, missing‐indicator by treatment group; PIT, probability imputation technique; UA, unadjusted analysis; WI, weighted overall mean imputation; WIT, weighted mean imputation by treatment group; WM, weighted missing‐indicator overall; WMT, weighted missing‐indicator by treatment group.

Unconditional mean imputation substitutes each missing value with the overall average of the observed values of the incomplete variable (covariate). By contrast, conditional mean imputation substitutes each missing value with the mean that is estimated conditional on the specific subgroup to which the individual with missing belongs. PIT is, in fact, conditional mean imputation for binary covariates. In the reviewed articles, unconditional mean imputation and conditional mean imputation were called overall mean imputation (I) and mean imputation by treatment group (IT), respectively. When a weighted analysis (with different weights for complete cases (CC) and IC) was performed after an imputation method, this method was referred to as weighted imputation, giving two new methods: weighted overall mean imputation (WI) and weighted mean imputation by treatment group (WIT). Weighting was performed as follows: CC were given a weight *ω*_*i*_ = 1 and cases with missing baseline were given a weight *ω*_*i*_ = 1 − ρ^2^, where ρ is the correlation between the outcome and the baseline, and was estimated using only CC. The factor 1 − ρ^2^ is the ratio of unexplained outcome variance for the CC relative to the unexplained variance for the IC. So this weighting amounts to weighting each case by its inverse variance, which is known to be more efficient than unweighted analysis (see, eg, Searle and Pukelsheim^28^) and was indeed shown to be able to improve efficiency of the estimated treatment effect (especially for ρ ≥ 60%).^4^, ^21^

The missing‐indicator method replaces the missing values of a variable with a fixed value and adds a dummy indicator to the analysis model to indicate whether the value of that variable is missing and thus imputed (1) or not (0) for an individual. Given the addition of this missing‐indicator, it does not matter which fixed value is used to impute, for instance, 0 value^6^ or the overall mean value.^4^, ^21^ But it still matters (as shown in section 3.3 of this study) whether the same value is imputed for all individuals or a distinct value per treatment group. The analysis could subsequently be either weighted (WM for weighted missing‐indicator) or unweighted (M for missing‐indicator). Weights in WM were the same as in WI and WIT above.

MI proceeds in two stages: first, the imputation stage, and second, the analysis and pooling stage. In the imputation stage:

1. the imputation model (with a set of parameters η) is defined as a conditional distribution of the incomplete variable (covariate in this article), conditional on the complete variables (in this article, treatment and also outcome), producing a set of parameter estimates $\hat{\eta }$ based on CC only;
2. a new set of parameter values η^*^ is randomly drawn from the posterior predictive distribution of η (using the standard noninformative prior, such as a uniform distribution, which assigns equal a priori weight of 1 to every possible value of the parameters, as in section 6.7 of Enders^9^ and section 3.2.2 of Van Buuren^13^);
3. η^*^ is used with the imputation model to obtain the posterior predictive distribution of the incomplete variable;
4. missing values in the incomplete variable are replaced with values randomly drawn (generated) from the posterior predictive distribution in step (3), producing one completed dataset; and
5. steps (2) to (4) are repeated several times to obtain several completed datasets. Note that, as suggested previously,^7^, ^13^ the random draws in step (2) and (4) are necessary to produce sufficient uncertainty (parameter uncertainty and inherent prediction error) in the imputed values.

In the analysis and pooling stage, first, the analysis of interest (eg, linear regression of the outcome on the covariate and the treatment in the complete dataset) is performed on each completed datasets. Each analysis produces the estimate of interest with its variance, resulting in multiple estimates and respective variances, corresponding to the number of completed datasets. Second, these estimates and their variances are then pooled to produce the overall estimate and its variance. Consider ${\hat{\beta }}_{1i}$ as the estimate of β_1_ from the *i*th completed dataset and $W_{i}={\mathrm{SE}}_{i}^{2}$ its variance, with SE_*i*_ its standard error (SE). The pooled estimate of β_1_ is calculated as ${\hat{\beta }}_{1}=1/m\sum_{i}^{m}{\hat{\beta }}_{1i}$ with as total variance *T*_var_ = *W* + *B*(1 + 1/*m*) and SE $\mathrm{SE}=\sqrt{T_{\mathrm{var}}}$; where $W=1/m\sum_{i}^{m}W_{i}$, $B={\left(m-1\right)}^{-1}\sum_{i}^{m}{\left({\hat{\beta }}_{1i}-{\hat{\beta }}_{1}\right)}^{2}$, and *m* the total number of completed datasets. *W* is the average within‐imputation variance, which measures the uncertainty in ${\hat{\beta }}_{1}$ that would have resulted had the data been complete. *B* is the between‐imputation variance, which measures the uncertainty due to the difference between the completed datasets.

MI produces unbiased estimates and appropriate SEs under MAR provided that the imputation model is correct and congenial with the analysis model. To be congenial, these two models need not be identical, but the imputation model must generate imputations that produce the major features of the data that are the focus of the analysis.^29^ For example, if the interest of the analysis is to estimate means, standard deviations, and the parameters of a linear regression model, then the imputation model needs only to preserve the means, variances, and covariances among the variables that will be analyzed.^30^ Thus, to ensure congeniality, it is recommended that the imputation model incorporates at least all variables (including the outcome) to be used in the analysis model. For example, if the analysis model contains interactions, the imputation model should also include them. This would mean creating the product variables before doing the imputation, and then imputing these along with the original variables.^29^ MI was performed overall (MIO) and separately by treatment group (MIT), with Groenwold et al^6^ using only overall MI and Sullivan et al^21^ using both approaches. For MIO, the imputation model was the distribution of the covariate conditional on the outcome and the treatment. For MIT, the imputation model was the distribution of the covariate conditional on the outcome only, performed within each group.

The likelihood‐based estimation of a linear mixed model (LMM) is a common alternative to MI for handling missing data in a multivariate outcome or in a repeatedly measured single outcome, here: for handling missingness of the baseline outcome measurement. Based on the multivariate normal distribution, this approach incorporates all observed information on the multiple outcomes or the repeated measures of an outcome to produce estimates that are valid under a MAR assumption. No explicit imputation is involved. So if the covariate is a baseline recording of the outcome (as is the case in articles P2, P3, and P4) it can be treated as a repeated measure in a LMM (as is the case in articles P2 and P4) under the assumption that the repeated measurements (ie, the covariate and the outcome) are jointly bivariate normally distributed and allowing for fixed effects of time, treatment, and time‐by‐treatment interaction. Due to the randomization, the treatment effect is set at zero at baseline and the effect of interest is the time‐by‐treatment interaction effect. Within‐subject correlation due to repeated measures is accounted for through the specification of a covariance structure. Several authors (including Sullivan et al^21^ in this review) have suggested the unstructured covariance matrix, which allows heterogeneity of variance over time and of correlation over pairs of time points. This covariance matrix can be easily prespecified, involves minimal power loss compared with more parsimonious choices even with as many as 5 to 10 repeated measures, except in very small samples,^11^ ^, p 53,54,^ ^31^ and guarantees that estimates are approximately identical to and slightly more efficient than those produced by a comparable MI procedure.^11^ MI is comparable to LMM (in procedure) when, as LMM, MI also assumes multivariate normality and MAR data, and incorporates into the imputation model all the variables used by LMM (including all repeated measurements and other variables predictive of missingness if available). Two LMM approaches were used in P2 and one in P4: for P2, LMM with and without missing‐indicator (LMMM and LMMS, respectively); and for P4, LMMS. For both approaches, missing values were not imputed; and LMMM only added an indicator of missingness to the model. Remember that the LMM approach is only feasible for missingness on the outcome, or on a baseline recording of the outcome, when the baseline is treated as a repeated outcome measure, not as a covariate.

Except for LMM and UA, the analysis model used in all the articles in Tables 1 and 2 was linear regression of the outcome on treatment and covariate without an interaction term.

#### 2.2.4 Comparison of methods for handling missingness

Schemper and Smith^20^ compared PIT to UA and CCA, and found that PIT preserved the correct type I error rate, provided that the incomplete covariate was independent of treatment. In general, PIT yielded more power than UA and CCA. However, the power of PIT was lower than the power of UA for the scenarios with missingness rate of 60%; and hence, it was advised not to use PIT in such situations. PIT was shown to have more power for situations with the covariate missing under MAR (missingness dependent on treatment only) than for situations with the covariate missing under MNAR (missingness dependent on the covariate itself only), because in the latter situations, unlike in the former, PIT leads to biased estimates of the covariate effect. Biased estimates of the covariate effect lead to increased residual variances, which reduce the power of all tests (for the treatment effect and covariate effect) in the model, even in randomized trials. PIT was said to produce unbiased treatment effect estimates when the covariate is uncorrelated with treatment (as in all randomized trials), regardless of the missingness types described above.

White and Thompson^4^ compared UA, CCA, I, IT, WI, M, WM, LMMS, and LMMM. They found that all the methods produced unbiased treatment effect estimates, irrespective of the missingness types. This was because the covariate was uncorrelated with treatment (because of randomization) and missingness was also uncorrelated with treatment (under MCAR missingness was totally independent, and under MNAR missingness depended only on the covariate) and so the missingness could not bias the estimation of the treatment effect. Almost any method was more precise than CCA; and LMMM, followed by LMMS, were slightly better than other methods. CCA was more precise than UA only for scenarios where the baseline covariate was strongly correlated with the outcome. Imputation per treatment group was shown to underestimate SEs and was therefore discouraged. Weighted approaches were shown to be more efficient than unweighted ones, and were advised, especially if the baseline covariate is correlated at least 0.6 with the outcome.

Though White and Thompson^4^ did not implement MI, they, however, questioned its suitability in RCTs. They argued that the use of MI to impute a missing covariate would require the inclusion of the outcome in the imputation model, as commonly advised for general regression models. In a randomized trial, this would seem to compromise the principle that adjustment should be made only for prerandomized covariates, as these covariates are unaffected by treatment. In other words, using an outcome that has already been affected by treatment to impute a covariate measured pretreatment, would amount to transferring part of the treatment effect to the covariate; and adjusting for that covariate in the outcome analysis would then lead to underestimation of the treatment effect. For MI to produce unbiased results, the outcome and the treatment would have to be simultaneously included in the imputation model. However, this would still seem to violate the randomization procedure. As a solution, White and Thompson^4^ advocated the use of mean imputation and missing‐indicator method for practical purposes. They argued that LMM and MI were unnecessarily complex, with little benefit in RCTs, when compared with simpler methods.

Groenwold et al^6^ analyzed the performance of the missing‐indicator method (M) in randomized and nonrandomized trials. For this review, however, we only focus on the results drawn from randomized trials. The authors compared M with CCA and MI. For MI, imputation was performed overall (MIO), and the imputation model was a linear regression of the covariate (prerandomization score) on the outcome (postrandomization score) and treatment. The authors, unlike White and Thompson,^4^ did not discuss the risk of breaching the randomization principle when the outcome and treatment are included in the imputation model of a prerandomized covariate. They demonstrated that M, MIO, and CCA produced unbiased treatment effect estimates with reasonable coverages. However, CCA generated wider confidence intervals (CIs), reflecting its loss of statistical power due to reduced sample size. They concluded, as the focus was on M, that M was appropriate to handle missing baseline covariates in randomized trials, regardless of the missingness type (note, however, that the authors only studied MCAR, where missingness was totally independent, and MNAR, where missingness was dependent only on the covariate itself). They noted that when the missing‐indicator method is used in the analysis model to adjust for an incomplete covariate, the estimated association between the outcome and treatment is a weighted average of two associations: (1) the association between the treatment and outcome, adjusted for all covariates, using only CC; (2) the association between the treatment and outcome, adjusted only for complete covariates, using only cases for which the incomplete covariate was missing. In randomized trials (with missingness as described above in this paragraph), these two associations are unbiased, because the complete and incomplete covariates, and the missingness of the incomplete covariate, are all uncorrelated with treatment. Since the two associations are unbiased, their weighted average is also unbiased.

Sullivan et al^21^ analyzed all the missingness types and methods covered by White and Thompson.^4^ In addition, Sullivan et al^21^ evaluated the performance of MI, which was the focus of their study; as in White and Thompson,^4^ they also highlighted the violation of the randomization principle when MI of a prerandomization covariate uses the outcome and/or treatment in the imputation model. MI was performed overall (MIO) and per treatment group (MIT). For MIO, the imputation model included the outcome and treatment. For MIT, the imputation model included only the outcome. Sullivan et al^21^ found that MI produced unbiased treatment effect estimates and was more efficient than CCA and UA. MIO and MIT performed similarly, with the former being slightly more efficient than the latter. Since MI did not outperform the other methods studied as alternatives to CCA and UA, Sullivan et al^21^ concluded that MI should not be considered as the gold standard method for handling missing baseline covariates in randomized trials. They, like White and Thompson,^4^ advocated the use of simpler methods such as mean imputation combined with a missingness indicator, especially if baselines are not MCAR; Under MCAR, mean imputation without missingness indicator was also said to be a good approach.

### 2.3 Identifying gaps

Using Tables 1 and 2, gaps can be identified as those combinations of analysis model, missingness types and methods for handling missing data that were not explored in the reviewed articles. For instance, the first row of Tables 1 and 2 combined shows that the following scenario has not yet been investigated: an RCT with continuous outcome and binary baseline covariate, where missingness (defined separately as MCAR, MAR, and MNAR) of the covariate is handled with any method (eg, MI, missing‐indicator, and so on) other than PIT, UA, CCA, and LMM, since PIT, UA, and CCA have already been studied in P1, and in practice, LMM is commonly fitted if the covariate is a baseline recording of the outcome variable (although an LMM can also be used for other continuous covariates, as long as the LMM model allows the covariate to have a very different mean and variance than the outcome (see, eg, Carpenter and Kenward^11^).

## 3 SIMULATION STUDY WITH A CONTINUOUS OUTCOME AND MISSING BINARY COVARIATE

### 3.1 Introduction

We studied a new scenario in RCT to fill some of the gaps identified in the reviewed articles. This scenario has in common with P1 the case of a binary covariate (so the analysis model is *Y*_1_ = β_0_ + β_1_*T* + β_2_*Z*_0_ + ε based on first row of Table 1). But, unlike P1, in our study (1) we consider a wider range of simulation scenarios as presented in section 3.2; (2) in addition to the methods used in P1 (ie, PIT, UA, and CCA), we evaluate all other methods (eg, MI), except LMM, considered in P2, P3, and P4; and (3) the binary covariate is not necessarily measured prerandomization: we consider both, and clearly distinguish, scenarios with a prerandomization covariate and those with a postrandomization covariate so that in the latter, missingness on the covariate can be allowed to depend on treatment. We repeat here that the situation where the baseline covariate is measured after randomization (so that its missingness can depend on treatment) is unlikely in pharmaceutical trials, but is unfortunately sometimes encountered in RCTs in other domains, for instance, in mental and public health (see Kole‐Snijders et al,^25^ Brug et al,,^26^ and Peters et al^27^), and in cluster randomized trials (see Kraag et al^23^ and Slok^24^). Henceforth, in this study, the linear regression model above is replaced with *Y* = β_0_ + β_1_*T* + β_2_*Z* + ε (note that *Z*_0_ and *Y*_1_ are replaced with *Z* and *Y*, respectively); where *Y*, *T*, and ε are, respectively, the posttest outcome, treatment group, and standard normal error; and *Z* is a binary covariate which is measured pre‐ or post‐randomization, according to the missingness mechanism under study, but is never itself affected by treatment. The effect of interest is β_1_, defined as treatment effect; β_2_ is the covariate effect and was not the concern of this study. We note that this analysis model may seem restrictive (ie, with only one missing covariate) in practice. The use of this model is, however, motivated by the following: We are interested in the comparison of a wide range of methods that have been used and are still being used in literature, to find out which are best and which break down as missingness increases or becomes MNAR. We use a simple model for this comparison to keep things clear and transparent, and select promising vs inferior methods based on this, to be studied further for more complex scenarios/models (with more than one covariate).

### 3.2 Method

We conducted a simulation study under various conditions. The basic steps of the simulation design were as follows.

#### 3.2.1 Generating complete data

We first created complete data from a hypothetical RCT with 50:50 allocation to treatment (*T* = 1) and control (*T* = 0). Two total sample sizes were considered: small (n = 100) and large (n = 400). The covariate Z was Bernoulli distributed with *P*(*Z* = 1) = *P*(*Z* = 0). Four combinations of the treatment effect and covariate effect were considered, that is (β_1_, β_2_) = (1, 1); (1, 2); (2, 1); and (2, 2). The intercept β_0_ was always set to 0. The error ε was standard normally distributed.

#### 3.2.2 Generating incomplete data

We obtained incomplete data by creating missing values in *Z* from the complete data generated in the previous step. Missing values were created based on the missingness model logit{*Pr*(*R* = 1)} = α_0_ + α_1_*Z* + α_2_*T* + α_3_*Y* + α_4_*ZT*; where *R* = 1 if *Z* is observed, and *R* = 0 if *Z* is missing. Five missingness mechanisms were considered, that is, MCAR, MAR, MNAR1, MNAR2, and MNAR3, with their parameters as given in Table 3. For MCAR, the missingness was unrelated to all variables. For MAR, the missingness was related only to *T*. For MNAR1, the missingness was related only to *Z*. For MNAR2, the missingness was both related to *Z* and *T*. For MNAR3, the missingness was both related to *Z*, *T*, and *ZT*. For each missingness mechanism, α_0_ was calculated based on the missingness rates required. Three missingness rates were considered, that is, 20%, 40%, and 60%. To obtain these rates, the value of α_0_ was adjusted accordingly. For example, under MNAR1b (ie, MNAR1 with α_1_ = 2), setting α_0_ = 0.3862944, α_0_ = −0.5945349, and α_0_ = −1.405465, produced approximately 20%, 40%, and 60% missingness rates, respectively. The missingness rates 40% and 60% may be unrealistic for a baseline covariate. We, nevertheless, include them in order to investigate what happens to the missing data methods in situations of substantial missingness rates.

**TABLE 3:** An overview of the missingness mechanisms in this article with their parameter values, based on the missingness model ***logit***{***Pr***(***R =* 1**)} ***= α***_**0**_ ***+ α***_**1**_***Z + α***_**2**_***T + α***_**3**_***Y + α***_**4**_***ZT***; where *R* = 1 if Z is observed, and *R* = 0 if Z is missing

Parameters	MCAR	MAR	MNAR1		MNAR2		MNAR3	
–	–	–	a	b	a	b	a	b
α_0_ (intercept)	≠ 0	≠ 0	≠ 0	≠ 0	≠ 0	≠ 0	≠ 0	≠ 0
α_1_ (coefficient of *Z*)	0	0	0.5	2	0.5	2	0.5	2
α_2_ (coefficient of *T*)	0	0.5	0	0	0.5	0.5	0.5	0.5
α_3_ (coefficient of *Y*)	0	0	0	0	0	0	0	0
α_4_ (coefficient of *ZT*)	0	0	0	0	0	0	1	1

*Note*: (MCAR, MNAR1) can always apply and (MAR, MNAR2, MNAR3) can only apply when the covariate is measured after randomization (but before treatment).

Note that α_3_ was set to 0 for all missingness mechanisms, so that missingness of *Z* is always independent of *Y*, as it is unlikely that missingness of the covariate, *Z*, which is measured before the outcome, *Y*, depends on this outcome. Where α_2_ ≠ 0, we assumed that the covariate was unrelated to treatment due to randomization, but its missingness depended only, or also, on treatment. This may happen if participants are first randomized to treatment groups, and the covariate, Z, is measured postrandomization (see section 2.2.2). Where α_2_ =. 0.5 (odds ratio 1.65), and α_2_ = 2 (odds ratio 7.39), missingness on the covariate is moderately and very strongly related to treatment, *T*, respectively

Note that of all missingness scenarios above, only MNAR2 and MNAR3 introduce weak, respectively, stronger (for the values of α_1_ and α_2_ as given in Table 3) confounding, as expressed by an odds ratio (OR) unequal to one, between the covariate (*Z*) and treatment (*T*) within the CC sample, and likewise within the IC sample. In fact, it can be proven (see Table 4 in the supplements) that:

1. OR for CC=OR for IC = 1 if (α_1_ = 0 or α_2_ = 0) and α_4_ = 0
2. OR for CC ≠ 1 and OR for IC ≠ 1 if (α_1_ ≠ 0 and α_2_ ≠ 0) or if α_4_ ≠ 0

Thus, for MCAR (α_1_ = 0, α_2_ = 0, α_3_ = 0, α_4_ = 0), MAR (α_1_ = 0, α_2_ ≠ 0, α_3_ = 0, α_4_ = 0), and MNAR1 (α_1_ ≠ 0, α_2_ = 0, α_3_ = 0, α_4_ = 0), there is no confounding (ie, OR for CC=OR for IC = 1), but for MNAR2 (α_1_ ≠ 0, α_2_ ≠ 0, α_3_ = 0, α_4_ = 0) and MNAR3 (α_1_ ≠ 0, α_2_ ≠ 0, α_3_ = 0, α_4_ ≠ 0), there is confounding, that is (OR for CC=OR for IC ≠ 1), respectively (OR for CC ≠ OR for IC ≠ 1). Consequently, this means that the treatment effect estimate under MCAR, MAR, and MNAR1 should be unbiased (since *Z* is not a confounder), but can be biased (since Z is a confounder within the CC and within the IC subgroups) under MNAR2 and MNAR3 depending on the methods and the value of α's. For the ORs in each scenario under MNAR2 and MNAR3 see Table 5 in the supplements.

#### 3.2.3 Imputing the missing data

We imputed the missing values to produce the completed data to be used in the analysis, except for UA (remove *Z*) and CCA (remove IC) analysis. For each dataset, we implemented the following missing data methods: UA; CCA; overall mean imputation (I); mean imputation per treatment group (IT); weighted overall mean imputation (WI); weighted mean imputation per treatment group (WIT); overall missing‐indicator (M), missing‐indicator per treatment group (MT); weighted overall missing‐indicator (WM); weighted missing‐indicator per treatment (WMT); MI overall (MIO); and MI per treatment group (MIT).

For overall imputation (methods I, WI, M, WM, and MIO), the values used to impute the missing values were calculated and imputed across treatment groups. For I, WI, M, and WM, for example, the missing values were always replaced with the mean of all the observed covariate values, irrespective of treatment group. For imputation per treatment group (methods IT, WIT, MT, WMT, and MIT), the values used to impute the missing values were calculated and imputed within each treatment group. For IT, WIT, MT, and WMT, for example, the missing values were always replaced with the mean of all observed covariate values within each treatment group.

For MI overall, the imputation model included both *Y* and *T*. For MIT, the imputation model within each group included only *Y*. Within MI overall as well as within MIT, two MI approaches were used: (1) specifying logistic regression of Z on *Y* and *T* as imputation model and proceeding as described in section 2.2.3; and (2) imputing based on predictive mean matching (PMM), which is similar to (1) in the analysis and pooling stage, but slightly different in the imputation stage. The difference is that, for PMM: (i) predicted values for *Z* are generated for all cases, both those with *Z* missing and those with *Z* observed; (ii) for each case with missing *Z*, a set of cases is drawn from all cases with observed *Z* whose predicted values are close to the predicted value for the case with missing *Z*; and (iii) from those drawn cases, one is randomly drawn and its observed value is used to replace the missing value.

#### 3.2.4 Analyzing the completed data

We applied the analysis of interest (linear regression of *Y* on *T* and *Z*) on the completed data to produce the treatment effect estimate (${\hat{\beta }}_{1}$) and its SE, and 95% confidence interval (CI). For each method, the analysis of interest was applied on each dataset, overall and not by treatment group. For UA, the analysis model included *Y* and *T*, but not *Z*. For all the other methods, the analysis model included *Y*, *T*, and *Z*. The missing‐indicator method and weighting were as in White and Thompson.^4^

#### 3.2.5 Compare the missing data methods performances

We compared the performance of the methods using datasets generated under each of 192 distinct scenarios, obtained by crossing the design factors (treatment and covariate effects, total sample size, missingness mechanisms, and missingness rates) defined in sections 3.2.1 and 3.2.2.

We generated 1500 different datasets (runs) for each scenario. For each dataset, estimates of the treatment effect, its SE and 95% CI were obtained with each missing data method, resulting in 1500 values of ${\hat{\beta }}_{1}$, its SE and CI under each scenario condition and each missingness method. These values were subsequently used to compute the mean and SD of ${\hat{\beta }}_{1}$ and of the estimated SE, as well as the coverage of the CI. Note that the SD of ${\hat{\beta }}_{1}$ estimates the true SE of ${\hat{\beta }}_{1}$ against which the mean of the estimated SE can be compared with check bias in SE estimation. The results for each missing data method were compared with those from the complete data, which served as the gold standard or reference method (REF). Thus, five performance criteria were calculated as follows. Consider that the *i*th simulated dataset (*i* = 1 to *s*) produces a treatment effect estimate ${\hat{\beta }}_{1i}$ with ${\hat{\mathrm{SE}}}_{i}$ and CI_*i*_; and *K*_*i*_ = 1 if β_1_ ∈ CI_*i*_ or *K*_*i*_ = 0 otherwise. Compute ${\bar{\hat{\beta }}}_{1}=\sum_{i}^{s}{\hat{\beta }}_{1i}/s$; $\mathrm{Var}\left({\hat{\beta }}_{1}\right)=\sum_{i}^{s}{\left({\hat{\beta }}_{1i}-{\bar{\hat{\beta }}}_{1}\right)}^{2}/\left(s-1\right)$; $\bar{\hat{\mathrm{SE}}}=\sum_{i}^{s}{\hat{\mathrm{SE}}}_{i}/s$;$\;\mathrm{Var}\left(\hat{\mathrm{SE}}\right)=\sum_{i}^{s}{\left({\hat{\mathrm{SE}}}_{i}-\bar{\hat{\mathrm{SE}}}\right)}^{2}/\left(s-1\right)$. The performance criteria were as follows:

- Bias of ${\hat{\beta }}_{1}$: $\text{Bias}\left({\hat{\beta }}_{1}\right)={\bar{\hat{\beta }}}_{1}-\beta_{1}$;
- Relative precision (RP) of ${\hat{\beta }}_{1}$ resulting from missingness method Meth (eg, CCA) compared with REF: $\mathrm{RP}\left({\hat{\beta }}_{1}\right)=\left\{{\mathrm{Var}}_{\mathrm{REF}}\left({\hat{\beta }}_{1}\right)/{\mathrm{Var}}_{\text{Meth}}\left({\hat{\beta }}_{1}\right)\right\}\times 100$;
- Coverage of 95% CI = $\text{Coverage}\left(95\%\mathrm{CI}\right)=\left(\sum_{i=1}^{s}K_{i}/s\right)\times 100$;
- Relative bias (RB) of $\hat{\mathrm{SE}}$ of ${\hat{\beta }}_{1}$: $\mathrm{RB}\left(\hat{\mathrm{SE}}\right)=\left\{\left(\bar{\hat{\mathrm{SE}}}/\sqrt{\mathrm{Var}\left({\hat{\beta }}_{1}\right)}\right)-1\right\}\times 100$;
- Relative precision of $\hat{\mathrm{SE}}$ of ${\hat{\beta }}_{1}$ from Meth compared with REF: $\mathrm{RP}\left(\hat{\mathrm{SE}}\right)=\left\{{\mathrm{Var}}_{\mathrm{REF}}\left(\hat{\mathrm{SE}}\right)/{\mathrm{Var}}_{\text{Meth}}\left(\hat{\mathrm{SE}}\right)\right\}\times 100$

Simulations were conducted in R 3.3.3. MI was performed using the MICE package version 2.30. MI is commonly implemented according to the rule of thumb that the number of imputations (completed datasets based on one incomplete dataset), *m*, should at least be equal to the percentage of missingness.^21^ We used *m* = 100 in this study. The computer processor used was IntelCorei5‐6500 cpu@3.20ghz 3.20GHz.

### 3.3 Results

We compare the methods, first per performance criterion, and then summarize this comparison per missingness scenario. We start by presenting the results of sample size n = 100 and the a‐scenarios in Table 3. Subsequently, we discuss the effect of increasing the sample size from 100 to 400, and the difference between a‐ and b‐scenarios in Table 3. In the sequel, the label non‐MNAR3a will be used as an abbreviation for “all missingness mechanisms other than MNAR3a”.

**FIGURE 1:**
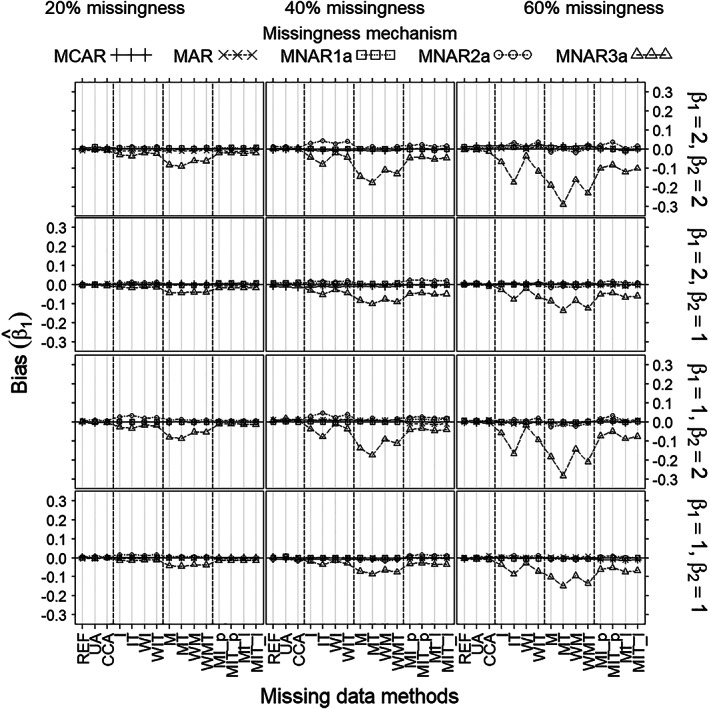
Bias of the treatment effect estimate (${\hat{\beta }}_{1}$) (*Y*‐axis) as a function of missingness method (*X*‐axis), for each scenario as defined by missingness rate (left/middle/right columns), missingness mechanism (curves), true treatment effect (β_1_) and covariate effect (β_2_) (rows), and sample size 100. Note that (MCAR, MNAR1a) can always apply and (MAR, MNAR2a, MNAR3a) can only apply when the covariate is measured after randomization (but before treatment)

Concerning bias of the treatment effect estimates (Figure 1), all methods had no or negligible bias under all non‐MNAR3a mechanisms. Under MNAR3a, only UA, CCA, and overall mean imputation (I and WI) had no bias; all other methods showed bias, but the bias of the missing‐indicator methods (M, MT, WM, and WMT) was substantially larger than that of the other methods.

**FIGURE 2:**
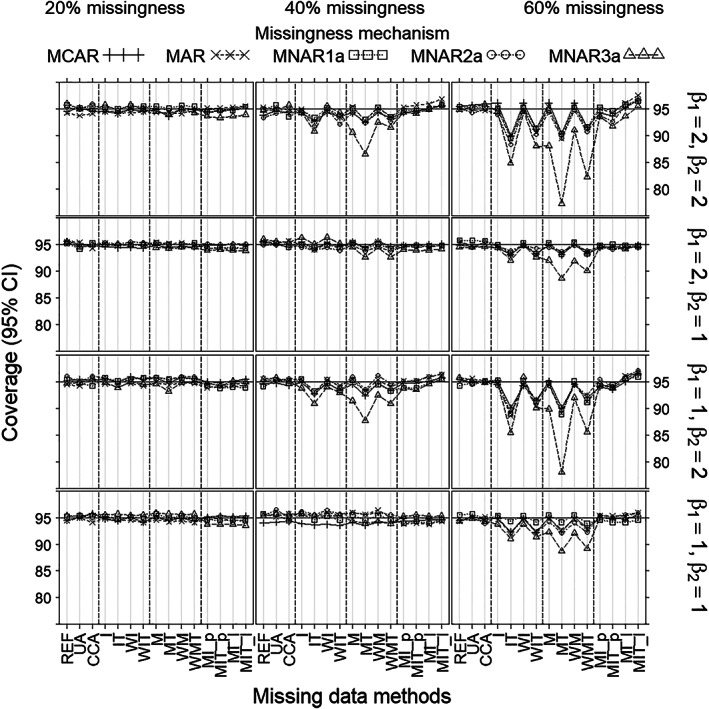
Coverage (%) of the 95% CI for the treatment effect estimate (*Y*‐axis) as a function of missingness method (*X*‐axis), for each scenario as defined by missingness rate (left/middle/right columns), missingness mechanism (curves), true treatment effect (β_1_) and covariate effect (β_2_) (rows), and sample size 100. Note that (MCAR, MNAR1a) can always apply and (MAR, MNAR2a, MNAR3a) can only apply when the covariate is measured after randomization (but before treatment)

For coverage (Figure 2), there was no noticeable difference between all non‐MNAR3a mechanisms. Under non‐MNAR3a, all methods except mean imputation per treatment (IT, WIT), and the missing‐indicator method per treatment (MT, WMT), provided coverages close to 95% in all scenarios; methods (IT, WIT) and methods (MT, WMT) showed substantial undercoverage when the missingness rate was 60% combined with strong covariate effect (β_2_ = 2). Under MNAR3a, only mean imputation per treatment (IT, WIT) and all versions of the missing‐indicator method (M, MT, WM, WMT) showed undercoverage when missingness was at least 40%. This was worst for the missing‐indicator methods, especially for strong covariate effect (β_2_ = 2).

**FIGURE 3:**
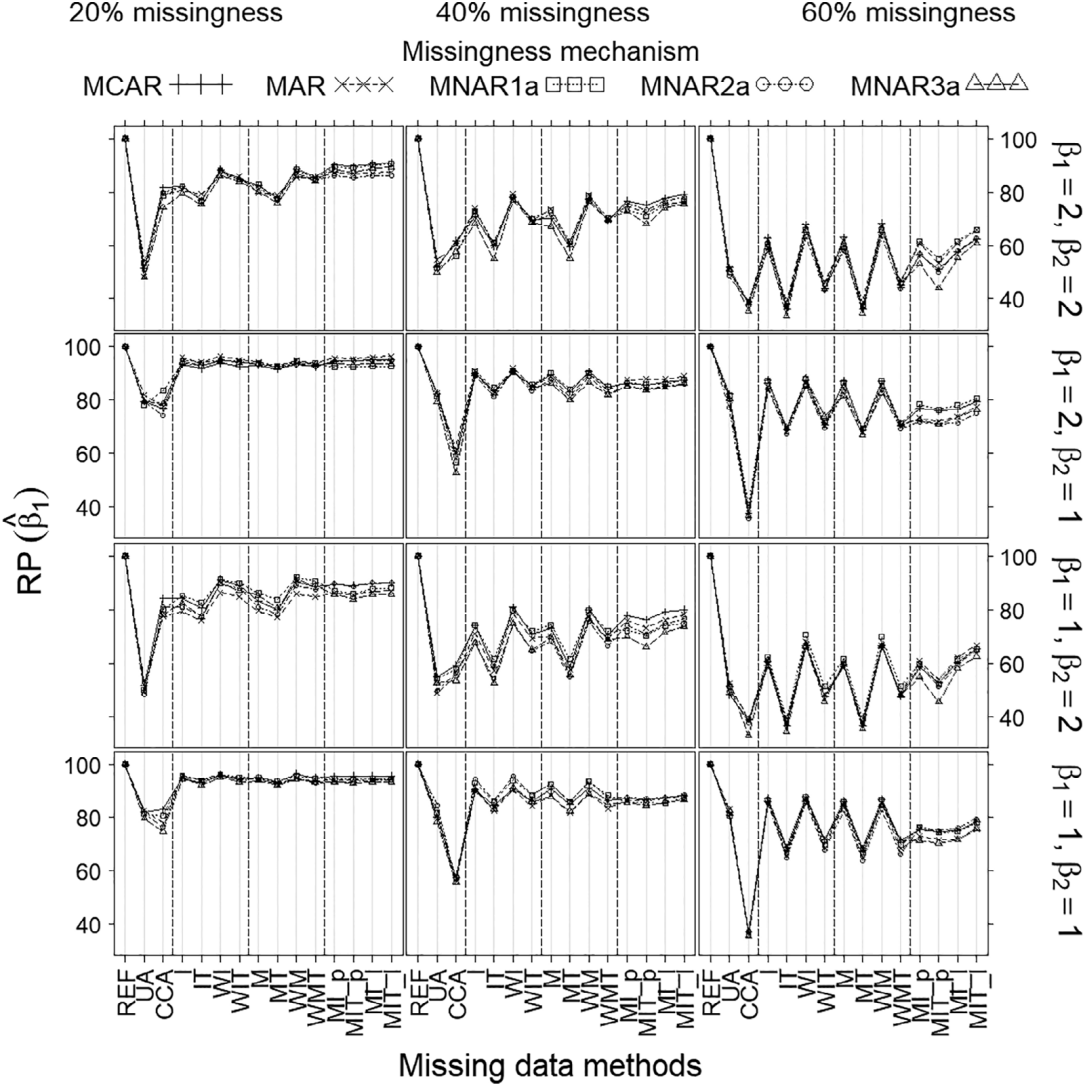
Relative precision of treatment effect estimate (${\hat{\beta }}_{1}$) (*Y*‐axis) as a function of missingness method (*X*‐axis), for each scenario as defined by missingness rate (left/middle/right columns), missingness mechanism (curves), true treatment effect (β_1_) and covariate effect (β_2_) (rows), and sample size 100. Note that (MCAR, MNAR1a) can always apply and (MAR, MNAR2a, MNAR3a) can only apply when the covariate is measured after randomization (but before treatment)

With respect to RP of the treatment effect estimates (Figure 3), which is the variance of the estimated treatment effect for REF (complete data) divided by the variance of the estimated treatment effect for the missing data method at hand, there was no noticeable difference between the missingness mechanisms, only between the methods. More specifically, UA was the least precise method when the missingness rate was 20% or 40% and the covariate effect was strong (β_2_ = 2). CCA was the least precise method when the missingness rate was 60%, or 40% and the covariate effect was modest (β_2_ = 1). Imputations overall were more precise than imputations per treatment, although the difference was negligible for MI. Weighted methods were slightly more precise than methods without weights. Mean imputation overall (I, WI) and the missing‐indicator method overall (M, WM) were most precise, in general. MI was as precise as the best methods only when the missingness rate was 20%. For all methods except UA, precision decreased with increased missingness rate; and for all methods except CCA, precision decreased with increased covariate effect.

**FIGURE 4:**
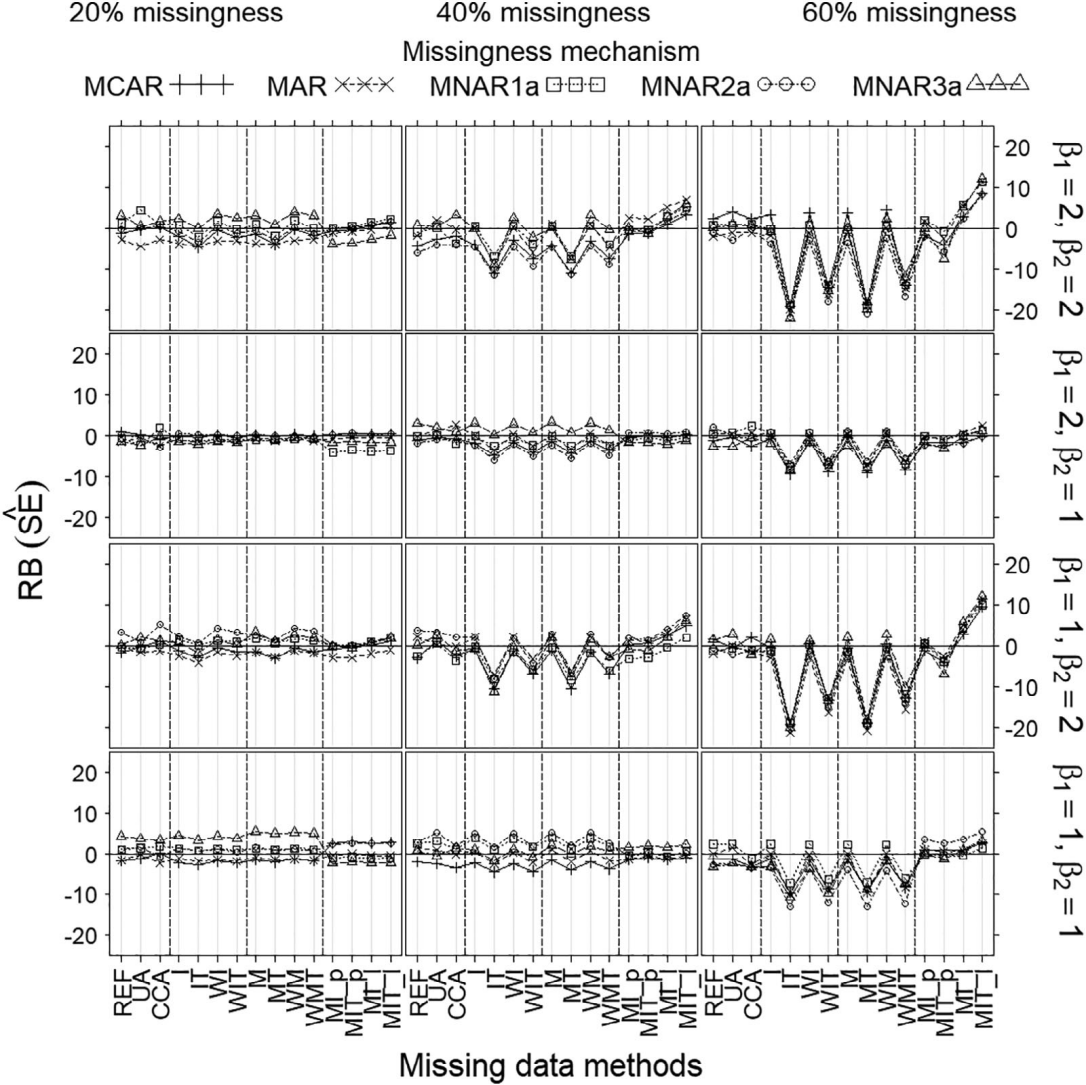
Relative bias of estimated standard error ($\hat{\mathrm{SE}}$) (*Y*‐axis) as a function of missingness method (*X*‐axis), for each scenario as defined by missingness rate (left/middle/right columns), missingness mechanism (curves), true treatment effect (β_1_) and covariate effect (β_2_) (rows), and sample size 100. Note that (MCAR, MNAR1a) can always apply and (MAR, MNAR2a, MNAR3a) can only apply when the covariate is measured after randomization (but before treatment)

Regarding RB of the estimated SE (Figure 4), which for each method is the difference between the estimated SE (model‐based SE) and the empirical SE divided by the empirical SE, there was no noticeable difference between the missingness mechanisms. Methods differed noticeably only for 60%, and for the combination of 40% missingness with strong covariate effect (β_2_ = 2), as follows: IT, WIT, MT, and WMT produced almost equal downward biases, while their respective counterparts (I, WI, M, and WM) had almost no bias; MIT_l showed some upward bias; whilst all other methods showed almost no bias. For methods with bias, biases increased with missingness rate and with covariate effect.

**FIGURE 5:**
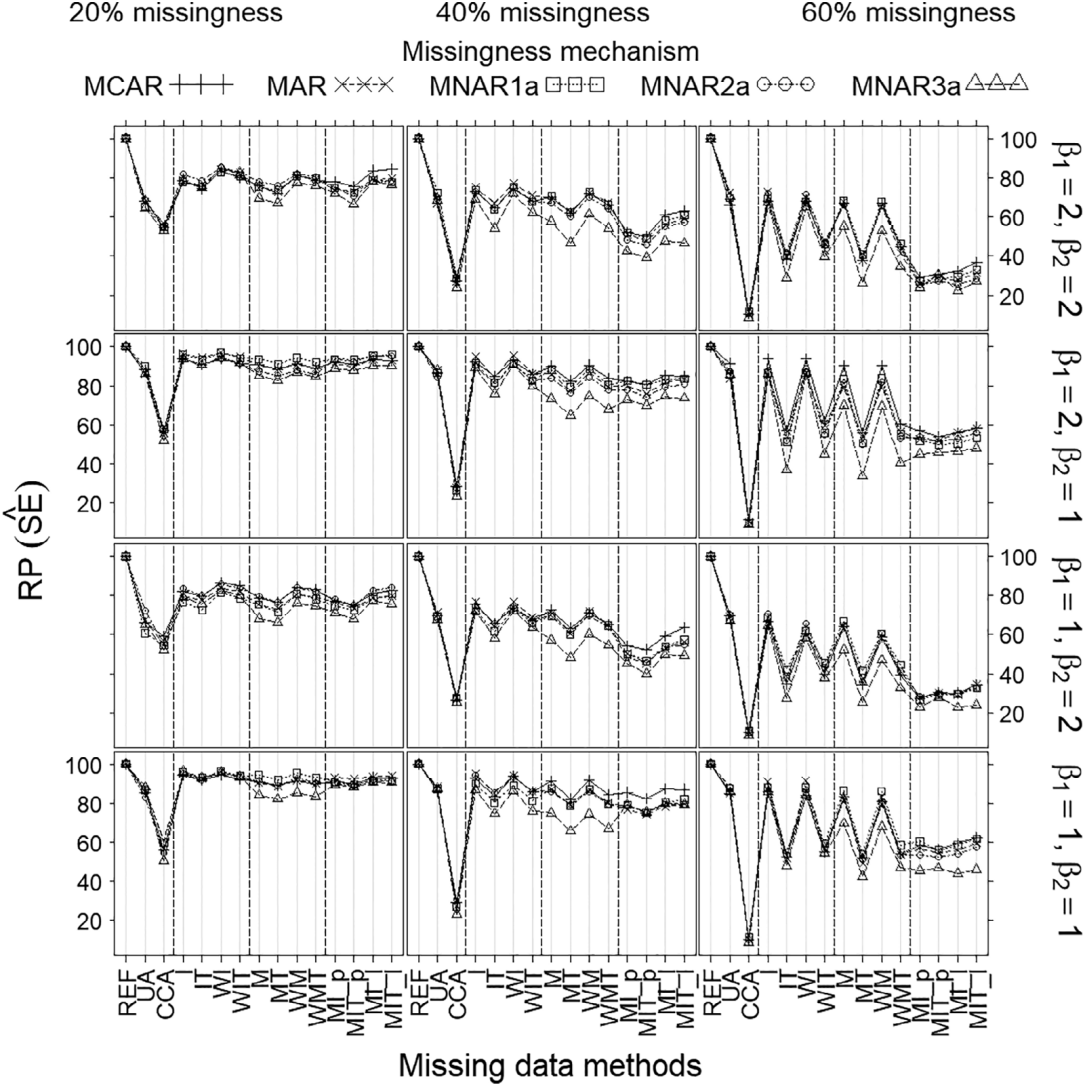
Relative precision of estimated SE ($\hat{\mathrm{SE}}$) (*Y*‐axis) as a function of missingness method (*X*‐axis), for each scenario as defined by missingness rate (left/middle/right columns), missingness mechanism (curves), true treatment effect (β_1_) and covariate effect (β_2_) (rows), and sample size 100. Note that (MCAR, MNAR1a) can always apply and (MAR, MNAR2a, MNAR3a) can only apply when the covariate is measured after randomization (but before treatment)

With respect to RP of the estimated SEs (Figure 5), which is the variance of the estimated SEs for REF (complete data) divided by the variance of the estimated SEs for the missingness method at hand, there was no substantial difference between the missingness mechanisms, except that MNAR3a showed slightly lower precisions compared with non‐MNAR3a when missingness was 40% or 60%. Methods differed as follows. UA was almost as precise as the best methods (I, WI, M, and WM), especially when missingness was 40% or 60%. CCA was least precise in all scenarios. Single imputations overall (I, WI, M, and WM) were more precise than their per treatment counterparts (IT, WIT, MT, and WMT): the differences were substantial when missingness was at least 40%. For MI, however, the differences between overall and per treatment versions were negligible. MI (all versions) was about as precise as the best methods (I, WI, M, and WM) only when missingness was 20%. For all methods except UA, precision decreased with increased missingness rate; and for all methods except CCA, precision decreased with increased covariate effect.

The effect of sample size on each of the five criteria (discussed above) was tested by increasing the sample size from 100 to 400. The results (Figures 6‐10 in the supplements) for each criterion were as follows. There was (1) little effect on bias of the treatment effect estimate; (2) negligible effect on coverage, apart from even lower coverages of the methods suffering from undercoverage in MNAR3a in Figure 2; (3) negligible effect on RP of the treatment effect estimate; (4) no noticeable effect on bias of the estimated SE of the treatment effect estimate, apart from the disappearance of the slight upward bias of MIT_l in Figure 4 (see panels with 60% missingness and covariate effect β_2_ = 2); and (5) substantial effect on RP of the estimated SE, but only for mean imputation and the missing‐indicator methods per treatment (IT, WIT, MT, WMT), which became almost as precise as their overall counterparts (I, WI, M, WM).

Increasing the effect of the covariate on its own missingness by increasing α_1_ from 0.5 to 2, corresponding to a‐ and b‐scenarios, respectively, gave no substantial differences, apart from bias of the treatment effect estimate under MNAR2 (Figures 11‐20 in the supplements), as follows. The mean imputation and MI methods showed a slight upward bias, and the missing‐indicator methods across treatments gave a slight downward bias, but only when missingness was 60% combined with covariate effect β_2_ = 2.

In summary, the pattern of results was nearly the same for all non‐MNAR3a scenarios, but different for the MNAR3a case. Pursuant to this, the comparison of the methods above is briefly done, first for non‐MNAR3a scenarios, and then for the MNAR3a scenario, as follows.

Under non‐MNAR3a scenarios, all methods gave no or negligible bias of the treatment effect estimate (${\hat{\beta }}_{1}$) (Figure 1). Bias of the estimated SE, however, was substantial for mean imputation per treatment and the missing‐indicator methods per treatment (IT, WIT, and MT, WMT) when missingness was at least 40%, and small or nonexistent for all other methods (Figure 4). Coverages (Figure 2) mirrored the pattern shown by bias of the estimated SE (Figure 4), being close to 95% wherever bias was negligible and lower wherever bias was substantial. Mean imputation and the missing‐indicator methods overall (I, WI, and M, WM), generally gave better precisions of ${\hat{\beta }}_{1}$ (Figure 3) and estimated SE (Figure 5) than all other methods; moreover, the differences increased with the missingness rate and the covariate effect. The precisions of MI were lower than those of methods (I, WI, M, WM) when missingness was at least 40%. The precisions of UA were the lowest for 20% missingness combined with strong covariate effect (β_2_ = 2); and the precisions of CCA were the lowest for at least 40% missingness.

Under MNAR3a, the missing‐indicator methods (M, MT, MW, WMT) gave substantial bias of ${\hat{\beta }}_{1}$; and mean imputation per treatment (IT, WIT) and MI showed some bias when missingness was at least 40%. For bias of the estimated SE, however, the differences between methods were the same as under non‐MNAR3a. Coverages for all methods except missing‐indicator were similar to those shown under non‐MNAR3a. The missing‐indicator methods suffered from substantial undercoverage under MNAR3a. In terms of precisions (of ${\hat{\beta }}_{1}$ and the estimated SE), the differences between methods under MNAR3a were similar to those under non‐MNAR3a.

## 4 EXTENSION TO TIME‐TO‐EVENT OUTCOME

We extended the simulation study to RCTs with a time‐to‐event outcome to evaluate the performance of all proposed missing data methods for such outcomes. This is important since nonlinear regression models (eg, the Cox model for time‐to‐event outcome) behave differently from linear regression models for a continuous outcome concerning estimation of treatment effects even when there is no missingness.^32^, ^33^, ^34^ For example, in nonlinear (unlike in linear) regression models, unadjusted (unconditional) and adjusted (conditional) treatment effect estimates differ numerically and are interpreted differently.^34^ Here we are interested in the conditional treatment effect since all the missing data methods (except UA) are based on covariate adjustment in the analysis model. Because of this and for simplicity, UA is not considered in this extension.

Except for the outcome, we used the same simulation setup (as in Section 3) to generate complete data as well as missing data. Survival times (*X*) were generated from a Weibull distribution: *h*_*X*_(*x*) = λ_*X*_*kx*^*k* − 1^exp(β_1_*T* + β_2_*Z*), where λ_*X*_ and *k* are the scale and shape parameters, respectively. Random censoring times were generated from a Weibull distribution with the same shape parameter: *h*_*C*_(*x*) = λ_*C*_*kx*^*k* − 1^. The parameter values used were λ_*X*_ = 0.002; *k* = 1 (as in White and Royston^35^); and λ_*C*_ = 0.0055 (corresponding to 50%; 41%; 41%; 33% censoring if (β_1_, β_2_) = (1, 1); (1, 2); (2, 1); (2, 2), respectively. The analysis model of interest used was the Cox proportional hazards (PH) regression model *h*_*X*_(*x*| *T*, *Z*) = *h*_0_(*x*)exp(β_1_*T* + β_2_*Z*) with *h*_0_(*x*) as baseline hazard.^35^, ^36^ Using this model, all the methods were implemented as described in section 3.2, except for MI. The imputation model for MI overall included *T*, *X*, and *S* (event indicator: 1 for event, 0 for censored observations).^35^ For MI per treatment (MIT), the imputation model within each group included only *X* and *S*. Using the five performance criteria described in section 3.2.5, all the methods were compared under all the missingness scenarios described in Table 3. Below we only present the key results for scenarios with sample size 100 (Figures 21‐25 in the supplements). The other results are provided in the supplements too (Figures 26‐30).

Like the case of a continuous outcome, the pattern of results under non‐MNAR3a was different from that under MNAR3a. In addition, there was no noticeable difference between non‐MNAR3a mechanisms, only between the methods, as discussed below.

Under non‐MNAR3a mechanisms, only CCA and MI per treatment (MIT_p, MIT_l) showed no or negligible bias for treatment effect estimates (Figure 21 in supplement). All other methods (I, IT, WI, WIT, M, MT, WM, WMT, MI_p, MI_l) had substantial downward bias, but only when the covariate effect was strong (β_2_ = 2), or when there was 60% missingness. Under MNAR3a, this pattern was the same, but the bias was larger. These results differ a bit from those for continuous outcomes (Figure 1) with respect to the various simple imputation methods and overall MI, but confirm the unbiasedness of CCA and MI per treatment.

Only CCA and MI gave coverage close to 95% under non‐MNAR3a (Figure 22 in supplement). Mean imputation and missing‐indicator methods generally showed acceptable coverage when β_2_ = 1, but undercoverage when β_2_ = 2. Under MNAR3a, coverage for CCA and MI was similar to the non‐MNAR3a cases. The other methods showed even worse undercoverage under MNAR3a as compared with non‐MNAR3a. These results differ from those for continuous outcomes in that serious undercoverage there only occurred under MNAR3. We get back to this difference in the discussion section.

For RP of ${\hat{\beta }}_{1}$ (Figure 23 in supplement), only CCA behaved as in the case of continuous outcome (Figure 3) by having lower precision than REF in all scenarios. Generally, imputation methods were about as precise as REF, except for lower precision of imputation per treatment methods under 60% missingness. The RB of the estimated SE (Figure 24 in supplement) for all methods was generally as in the case of a continuous outcome (Figure 4), except for slightly bigger upward bias of MI overall (MI_p, MI_l). With respect to RP of the estimated SE (Figure 25 in supplement), only precision for CCA and MI was clearly at least 10% below that of REF in all scenarios. A difference with the case of a continuous outcome (Figure 5) is that the simple imputation methods now tend to have higher precision than REF in case of a strong covariate effect or a high missingness rate. This finding will also be discussed in the following section.

## 5 DISCUSSION

In this article, we investigated the problem of missing covariates in RCTs. We first conducted a review of studies on methods to handle missing covariates in a RCT with a continuous outcome. The review revealed that the case with a covariate as a baseline measurement of the outcome had received considerable attention in the literature.^4^, ^6^, ^21^ However, the situation with a binary covariate had received only scant attention. Schemper and Smith^20^ performed a limited simulation study comparing only three methods: UA, CCA, and mean imputation by treatment. In the present study, we covered a wider range of simulation scenarios with more methods for handling missing data and more missingness mechanisms than in all the reviewed articles.

Our simulations showed that the pattern of results was very similar for all non‐MNAR3 scenarios, but different for MNAR3. This underlines the importance of investigating whether the covariate (*Z*) is missing based on a MNAR3 mechanism, before prescribing a method to deal with missingness of *Z*. MNAR3 means that missingness of *Z* is a nonadditive function of Z and treatment (*T*), and can therefore be detected as follows: Randomization ensures the absence of a treatment group difference on *Z*, and this holds for complete data as well as for MCAR, MAR, MNAR1 and MNAR2 missingness where *Z* and *T* have no or only additive effects on missingness. By contrast, it does not hold for MNAR3. So if CCA is performed with *Z* as outcome and *T* as predictor, ignoring the real outcome *Y*, then finding a treatment effect on *Z* indicates MNAR3.

For non‐MNAR3, the key finding is that all methods have no or negligible bias for the treatment effect estimate (${\hat{\beta }}_{1}$), but differ in other respects depending on the scenarios, as subsequently discussed. UA loses substantial precision when the covariate effect is strong. This makes sense because, when the covariate is strongly associated with the outcome, the residual outcome variance can be strongly reduced by adjusting for the covariate, thus also reducing the SE of the treatment effect. CCA loses substantial precision if the proportion of missingness is high (here at least 20%). This is also not surprising because the more cases are removed from the analysis, the more information and, hence, precision is lost. This agrees with all the reviewed articles.

Mean imputation overall (I, WI) and the missing‐indicator method overall (M, WM) are superior to their by treatment counterparts (IT, WIT, and MT, WMT) and give acceptable results in all respects. The by treatment methods lose precision, produce biased SE estimates and undercoverage, depending on the scenarios. Weighted methods are slightly more precise than unweighted methods only if the covariate effect is strong. This agrees with White and Thompson^4^ and Sullivan et al^21^: when the covariate effect is strong, the difference in residual outcome variances between cases with and without missingness is likely to be substantial. Weighting takes that into account by giving more weight to CC than to IC, thereby improving precision.

MI (all versions) performs roughly as well as mean imputation overall with or without missingness indicator: this holds in all respects except precision, which is noticeably lower for MI when missingness is very high. This is not surprising as MI, unlike single imputation methods, accounts for uncertainty due to imputation, resulting in increased variance. Single imputation methods, on the other hand, in general results in underestimated variance (overestimated precision). Furthermore, there is no substantial difference between MI overall and per treatment, although the former was slightly more precise in the majority of scenarios. This makes sense as long as the analysis model is correctly specified (as in this study). But if the analysis model were to be misspecified (as was the case in some scenarios studied by Sullivan et al^21^ for missingness on the outcome), MI per treatment might be beneficial. Finally, there is no noticeable difference between MI with logistic regression and MI with PMM either.

For MNAR3, the key finding is that only UA, CCA and mean imputation overall give no or negligible bias for the treatment effect estimate, and that the missing‐indicator method yields substantial bias and undercoverage depending on the scenarios. MI shows some bias only when the missingness proportion is high. In all other respects, results for all methods mirrored those produced under non‐MNAR3.

The bias of the missing‐indicator method under MNAR3 (when missingness depends on treatment (*T*) and treatment‐by‐covariate interaction (T*Z)), can be understood as follows: As argued by Groenwold et al,^6^ when the missing‐indicator method is used in the analysis model to adjust for an incomplete covariate, the estimated association between the outcome and treatment is a weighted average of two associations: (1) the association between the treatment and outcome, adjusted for all covariates, using only CC; (2) the association between the treatment and outcome, adjusted only for complete covariates, using only cases for which the incomplete covariate was missing (IC). Now, when missingness is driven by T*Z interaction, this induces an association between covariate (*Z*) and treatment (*T*) in both subgroups, the CC and the IC, making *Z* a confounder for estimating the treatment effect within each subgroup. In the subgroup of CC, the bias due to confounding is removed, since the confounder *Z* is fully observed and is included in the analysis model. However, in the subset of patients with missing covariate (IC), the estimate of the treatment effect is biased because the (unmeasured) confounder *Z* cannot be included in the model. Consequently, the weighted average of the treatment effect estimates from the CC and the IC subgroups is biased.

However, this explanation raises a new question, namely, why mean imputation gave much less bias than the missing‐indicator method under MNAR3, even though both methods come down to a weighted mean of CC and IC analyses and the latter (IC) suffers from confounding. The explanation for the much smaller bias of mean imputation is that, in a linear covariate effect model (which is the only option for a 0/1 covariate *Z*), persons with missing and thus mean imputed *Z* get much less weight in the analyses than persons with observed *Z* = 0 or *Z* = 1 (cf the linear contrast in polynomial trend analyses in regression). To test that explanation, we added as predictor to the analysis model the quadratic term *Z* ^2^ after using mean imputation because, given that *Z* has 3 distinct values after mean imputation (0, 1, mean imputed), combining Z and *Z* ^2^ gives a full account of possible effects of *Z* on *Y*, equivalently to using dummy coding of *Z* with 2 dummies, or to using the missing‐indicator method (which also has two predictors to represent the covariate, namely, Z itself and the missing indicator). Indeed, extending the analysis model after mean imputation with *Z* ^2^ gave the same bias and coverage as the missing‐indicator method under MNAR3 (results not shown).

Finally, concerning the bias of MI under MNAR3, that may be due to misspecification of the imputation model, which did not include the interaction (*TZ*). In fact, such inclusion is impossible if *Z* itself is missing.

We acknowledge that covariate missingness dependent on treatment (ie, our MNAR3, MNAR2, and MAR) can only occur if the baseline covariate is measured postrandomization. This practice is not considered acceptable in drug trials, but is unfortunately sometimes encountered in RCTs in other health domains, for instance, in mental and public health (see Kole‐Snijders et al,^25^ Brug et al,^26^ and Peters et al^27^), and in cluster randomized trials (see Kraag et al^23^ and Slok^24^). We therefore included this type of missingness as a relevant extension of the articles found in the scoping review. The results, especially for MNAR3, are a clear warning against measuring covariates after randomization and thus further support for a dominant practice in drug trials.

Regarding the choice of methods for handling missing data, the following recommendations can be given for the present case of a single binary covariate and a continuous outcome: UA if the covariate effect is weak and missingness is at least 60%, and CCA if missingness is low (less than 20%). The advantage of both methods is the ease of performing the analysis. Mean imputation overall (I, WI) is a valid method in all respects and can be used under all missingness mechanisms. The missing‐indicator method overall (M, WM) is also good in all respects, but only under non‐MNAR3 mechanisms. Furthermore, unless the covariate effect is very strong, imputation without weighting is preferable in practice for ease of implementation. Mean imputation per treatment with or without missing‐indicator are not advised because they are inferior in many respects. MI (preferably overall) is acceptable under non‐MNAR3, but not under MNAR3 because it shows some bias. MI per treatment is discouraged because it requires more work compared with MI overall. The choice between MI with logistic regression and MI with PMM should be a matter of convenience, as these methods provide similar results.

When comparing the simulation results in the case of time‐to‐event outcome to the case of continuous outcome in the present article, all methods except CCA and MI per treatment performed differently from how they performed for continuous outcome under non‐MNAR3. Specifically, only CCA and MI per treatment give no or negligible bias with good coverage for the treatment effect estimate (${\hat{\beta }}_{1}$) in all scenarios as in the case of a continuous outcome. MI overall performs similarly to their per treatment counterparts only for modest covariate effect. For strong covariate effect, they show some bias. This agrees with White and Royston.^35^

Mean imputation and missing‐indicator methods give substantial bias and undercoverage if the covariate effect (β_2_) is strong. This can be understood as follows: For generalized linear regression models (at least the logistic and Cox models), the treatment effect estimate (${\hat{\beta }}_{1}$) conditional on a covariate is always moved away from zero compared with the unconditional one.^32^, ^33^, ^37^ As the correlation between the outcome and covariate (and so the covariate effect) is weakened by replacing a missing Z with the mean, the methods shift from estimating the conditional effect of treatment (β_1_) to estimating the unconditional one (ie, without covariate Z) and, thus, produce bias toward zero for ${\hat{\beta }}_{1}$ in generalized linear models.^21^ For the same reason, mean imputation and missing‐indicator methods suffer from low coverage of the conditional treatment effect estimate wherever they have substantial downward bias.

Comparison of precisions in the presence of bias is tricky. Nonetheless, unlike for a continuous outcome where simple imputation methods are less precise than the REF method, they are for a time‐to‐event outcome almost as precise as REF for modest covariate effect and more precise than REF for strong covariate effect. This also makes sense: Not conditioning on a (nonconfounding) covariate in generalized linear models (at least logistic and Cox models) results in smaller SEs (and so higher precision) than conditioning.^21^, ^37^ Simple imputation methods reduce the covariate‐outcome correlation (and so the covariate effect) and thus have the same effect on SEs (precision) as when there is no conditioning on Z, but smaller.

Under MNAR3, all methods except simple imputation methods perform as under non‐MNAR3. Simple imputation methods give even worse bias and undercoverage under MNAR3 than under non‐MNAR3.

Regarding the choice of methods for dealing with missing data in a binary covariate for time‐to‐event outcome in the context of our simulation, the following recommendations can be given. CCA is a good method in terms of bias and coverage, but is acceptable in terms of precision only if missingness is low (less than 20%). Mean imputation overall (I, WI) and the missing‐indicator method overall (M, WM) are preferable to their counterparts per treatment (IT, WIT) and (MT, WMT). They are acceptable in all respects, but only under non‐MNAR3 if the covariate effect is modest (β_2_ = 1). MI per treatment (MIT_p, MIT_l) are the best methods in all respects and all scenarios. MI overall (MI_p, MI_l) are suitable and perform like their per treatment counterparts if the covariate effect is modest (β_2_ = 1).

This article is, and so the findings thereof are, limited to RCTs. But things will be different in observational studies which are beyond the scope of this article. Readers interested in the topic of missing predictors/covariates in observational studies can see White and Carlin,^5^ Qu and Lipkovich,,^14^ and Leyrat et al.^15^ Furthermore, this article is limited to RCTs with a continuous outcome and a missing binary covariate, with some results for time‐to‐event outcomes. However, scenarios with more covariates are common in practice. For example, a trial with a binary covariate and a continuous outcome which is measured both pre‐ and post‐randomization. A possible extension of the current study would then be to consider joint missingness of two or three of these variables, which would give rise to more missingness mechanisms to investigate. Another possible extension would be to consider binary instead of continuous outcomes, or to extend the present work on time‐to‐event data.

### DATA SHARING

The data (R‐code) that support the findings of this study are available from the corresponding author (mutamba.kayembe@maastrichtuniversity.nl) upon request.

## Supporting information


**Appendix S1**: Supporting Information

## Data Availability

The data (R‐code) that support the findings of this study are available from the corresponding author (mutamba.kayembe@maastrichtuniversity.nl) upon reasonable request.
