# Supplementary material for: Imputation of missing covariate in randomized controlled trials with a continuous outcome: Scoping review and new results
Source: Pharm Stat. 2020 Jun 8;19(6):840–60. doi: 10.1002/pst.2041 (PMC7687108; doi:10.1002/pst.2041)
Supplement: Supplementary file 1 — Appendix S1: Supporting Information [file PST-19-840-s001.docx]

**SUPPLEMENTARY TABLES AND FIGURES**

SUPPLEMENTARY TABLES

The proof of the confounding (or lack thereof) introduced between the covariate (Z) and treatment (T) within the CC sample, and likewise within the IC sample, can be given by filling in each cell of the T by Z cross-table for the CC sample and for the IC sample, first in terms of the probabilities implied by the missingness model in general, and then for a specific set of parameter values for that model, as follows.

Recall that the missingness model as given in section 3.2.2 of the manuscript is $logit\left\{ Pr\left( R=1 \right) \right\}=\alpha_{0}+\alpha_{1}Z+\alpha_{2}T+\alpha_{3}Y+\alpha_{4}ZT$; where R = 1 if Z is observed, and R = 0 if Z is missing. It follows therefore that:

$$Pr\left( R=0 \right)=\frac{1}{1+e^{\alpha_{0}+\alpha_{1}Z+\alpha_{2}T+\alpha_{3}Y+\alpha_{4}ZT}}$$

and

$$Pr\left( R=1 \right)=\frac{e^{\alpha_{0}+\alpha_{1}Z+\alpha_{2}T+\alpha_{3}Y+\alpha_{4}ZT}}{1+e^{\alpha_{0}+\alpha_{1}Z+\alpha_{2}T+\alpha_{3}Y+\alpha_{4}ZT}}$$

Now, filling in each cell of the cross-table the two probabilities above results in:

Table 4: Formulas for probability of incomplete cases (IC) and complete cases (CC) per cell, and for odds ratio (OR) within IC, and likewise within CC

| **Probability of IC ((Pr(R=0))** | | |
| --- | --- | --- |
|  | Z=0 | Z=1 |
| T=0 | $\frac{1}{1+e^{\alpha_{0}}}$ | $\frac{1}{1+e^{\alpha_{0}+\alpha_{1}}}$ |
| T=1 | $\frac{1}{1+e^{\alpha_{0}+\alpha_{2}}}$ | $\frac{1}{1+e^{\alpha_{0}+\alpha_{1}+\alpha_{2}+\alpha_{4}}}$ |
| OR | $\left( \frac{1}{1+e^{\alpha_{0}}}\times\frac{1}{1+e^{\alpha_{0}+\alpha_{1}+\alpha_{2}+\alpha_{4}}} \right)/\left( \frac{1}{1+e^{\alpha_{0}+\alpha_{2}}}\times\frac{1}{1+e^{\alpha_{0}+\alpha_{1}}} \right)$ | |
| **Probability of CC ((Pr(R=1))** | | |
|  | Z=0 | Z=1 |
| T=0 | $\frac{e^{\alpha_{0}}}{1+e^{\alpha_{0}}}$ | $\frac{e^{\alpha_{0}+\alpha_{1}}}{1+e^{\alpha_{0}+\alpha_{1}}}$ |
| T=1 | $\frac{e^{\alpha_{0}+\alpha_{2}}}{1+e^{\alpha_{0}+\alpha_{2}}}$ | $\frac{e^{\alpha_{0}+\alpha_{1}+\alpha_{2}+\alpha_{4}}}{1+e^{\alpha_{0}+\alpha_{1}+\alpha_{2}+\alpha_{4}}}$ |
| OR | $\left( \frac{e^{\alpha_{0}}}{1+e^{\alpha_{0}}}\times\frac{e^{\alpha_{0}+\alpha_{1}+\alpha_{2}+\alpha_{4}}}{1+e^{\alpha_{0}+\alpha_{1}+\alpha_{2}+\alpha_{4}}} \right)/\left( \frac{e^{\alpha_{0}+\alpha_{2}}}{1+e^{\alpha_{0}+\alpha_{2}}}\times\frac{e^{\alpha_{0}+\alpha_{1}}}{1+e^{\alpha_{0}+\alpha_{1}}} \right)$ | |

It follows from the OR formulas in Table 4 that:

1. OR for CC=OR for IC=1 (i.e. no confounding in either subgroup) if (α_1_=0 or α_2_=0) and α_4_=0
2. OR for CC≠ 1 and OR for IC ≠1 (i.e. confounding in both subgroups) if (α_1_≠0 and α_2_≠0), which is MNAR2, or if α_4_≠0, which is MNAR3

which in turn result in the OR for IC and for CC as given in the last paragraph of section 3.2.2 in the manuscript for MCAR, MAR, MNAR1, MNAR2 and MNAR3 as described in Table 3 of the manuscript. So there is confounding (OR≠1) only in scenarios under MNAR2 and MNAR3 with OR’s as given in Table 5.

Table 5: Odds ratio (OR) for incomplete cases (IC) and complete cases (CC) for each scenario under MNAR2a, b and MNAR3a, b.

|  | MNAR2a |  |  | MNAR3a |  |  |
| --- | --- | --- | --- | --- | --- | --- |
| P(R=0) | 20% | 40% | 60% | 20% | 40% | 60% |
| OR for IC | 0.96 | 0.94 | 0.94 | 0.39 | 0.44 | 0.53 |
| OR for CC | 0.96 | 0.94 | 0.94 | 1.06 | 1.20 | 1.43 |
|  | MNAR2b |  |  | MNAR3b |  |  |
| P(R=0) | 20% | 40% | 60% | 20% | 40% | 60% |
| OR for IC | 0.85 | 0.80 | 0.80 | 0.32 | 0.33 | 0.38 |
| OR for CC | 0.85 | 0.80 | 0.80 | 0.88 | 0.91 | 1.04 |
| P(R=0): proportion of missingness; OR: odds ratio; IC: Incomplete cases; CC: Complete cases. | | | | | | |

SUPPLEMENTARY FIGURES

Here, we provide the supplementary figures, which are not shown in the manuscript. The equation below is the missing data model used to generate the different missingness mechanisms with the corresponding parameters shown in Table A, which is Table 3 in the manuscript and provided below as a recall. The numbering of figures starts from six (i.e. Figure 6 and so forth), as a direct sequel to the five figures (i.e. Figures 1-5) contained in the manuscript.

Missingness model: $logit\left\{ Pr\left( R=1 \right) \right\}=\alpha_{0}+\alpha_{1}Z_{0}+\alpha_{2}T+\alpha_{3}Y_{1}+\alpha_{4}Z_{0}T$, where R = 1 if Z is observed, and R = 0 if Z is missing.

Table A (referred to as Table 3 in the manuscript): An overview of the missingness mechanisms with their parameter values.

| Parameters | MCAR | MAR | MNAR1 | | MNAR2 | | MNAR3 | |
| --- | --- | --- | --- | --- | --- | --- | --- | --- |
| - | - | - | a | b | a | b | a | b |
| α_0_ (intercept) | ≠ 0 | ≠ 0 | ≠ 0 | ≠ 0 | ≠ 0 | ≠ 0 | ≠ 0 | ≠ 0 |
| α_1_ (coefficient of *Z*) | 0 | 0 | 0.5 | 2 | 0.5 | 2 | 0.5 | 2 |
| α_2_ (coefficient of *T*) | 0 | 0.5 | 0 | 0 | 0.5 | 0.5 | 0.5 | 0.5 |
| α_3_ (coefficient of *Y*) | 0 | 0 | 0 | 0 | 0 | 0 | 0 | 0 |
| α_4_ (coefficient of *ZT*) | 0 | 0 | 0 | 0 | 0 | 0 | 1 | 1 |
| Note: (MCAR, MNAR1) can always apply and (MAR, MNAR2, MNAR3) can only apply when the covariate is measured after randomization (but before treatment). | | | | | | | | |

Scenarios with continuous outcome

Results under MCAR, MAR, MNAR1a, MNAR2a, and MNAR3a, for sample size 400


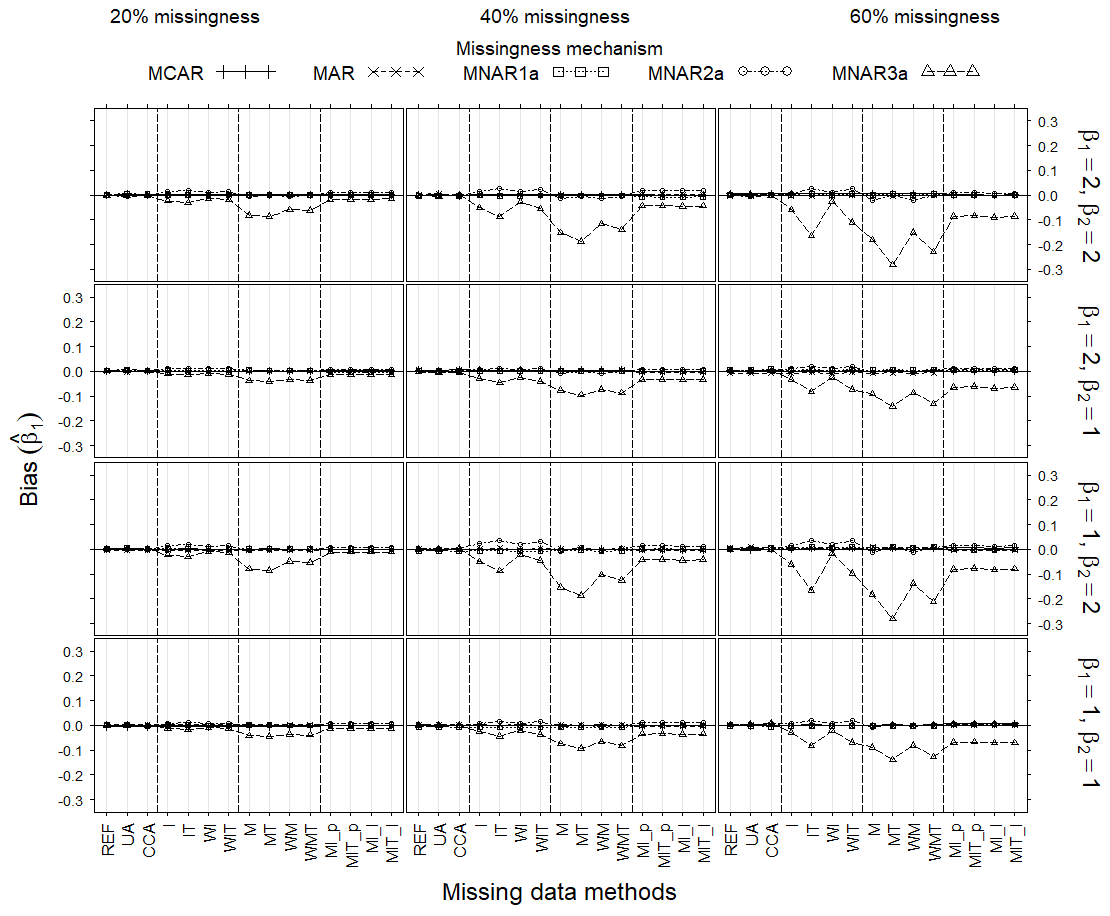


**Figure 6**. Bias of the treatment effect estimates ($Bias\left( \hat{\beta}_{1} \right)$) (Y-axis) as a function of missingness method (X-axis), for each scenario as defined by missingness rate (left/middle/right columns), missingness mechanism (curves), true treatment effect (β_1_) and covariate effect (β_2_) (rows), and sample size 400. Note that (MCAR, MNAR1a) can always apply and (MAR, MNAR2a, MNAR3a) can only apply when the covariate is measured after randomization (but before treatment).


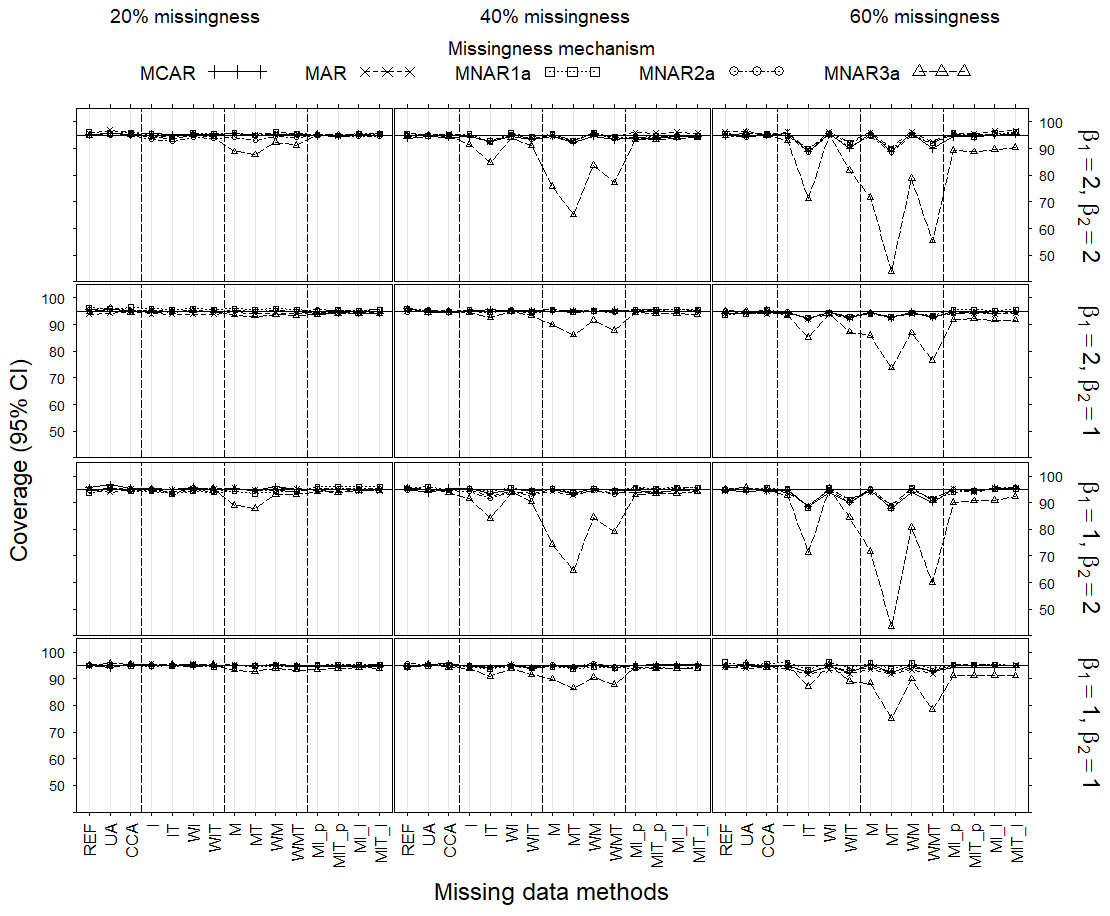


**Figure 7**. Coverage (%) of the 95% CI for the treatment effect estimate ($\hat{\beta}_{1}$) (Y-axis) as a function of missingness method (X-axis), for each scenario as defined by missingness rate (left/middle/right columns), missingness mechanism (curves), true treatment effect (β_1_) and covariate effect (β_2_) (rows), and sample size 400. Note that (MCAR, MNAR1a) can always apply and (MAR, MNAR2a, MNAR3a) can only apply when the covariate is measured after randomization (but before treatment).


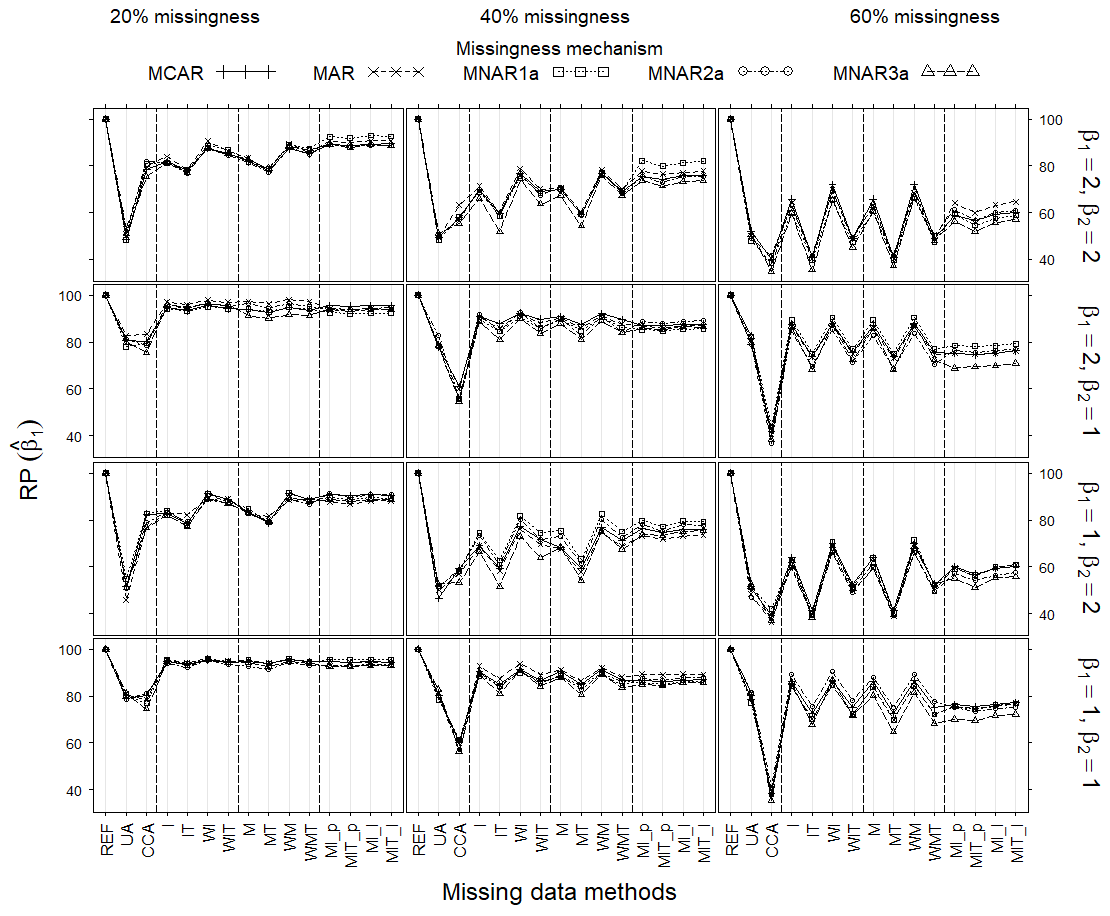


**Figure 8**. Relative precision (RP) of treatment effect estimate ($\hat{\beta}_{1}$) (Y-axis) as a function of missingness method (X-axis), for each scenario as defined by missingness rate (left/middle/right columns), missingness mechanism (curves), true treatment effect (β_1_) and covariate effect (β_2_) (rows), and sample size 400. Note that (MCAR, MNAR1a) can always apply and (MAR, MNAR2a, MNAR3a) can only apply when the covariate is measured after randomization (but before treatment).


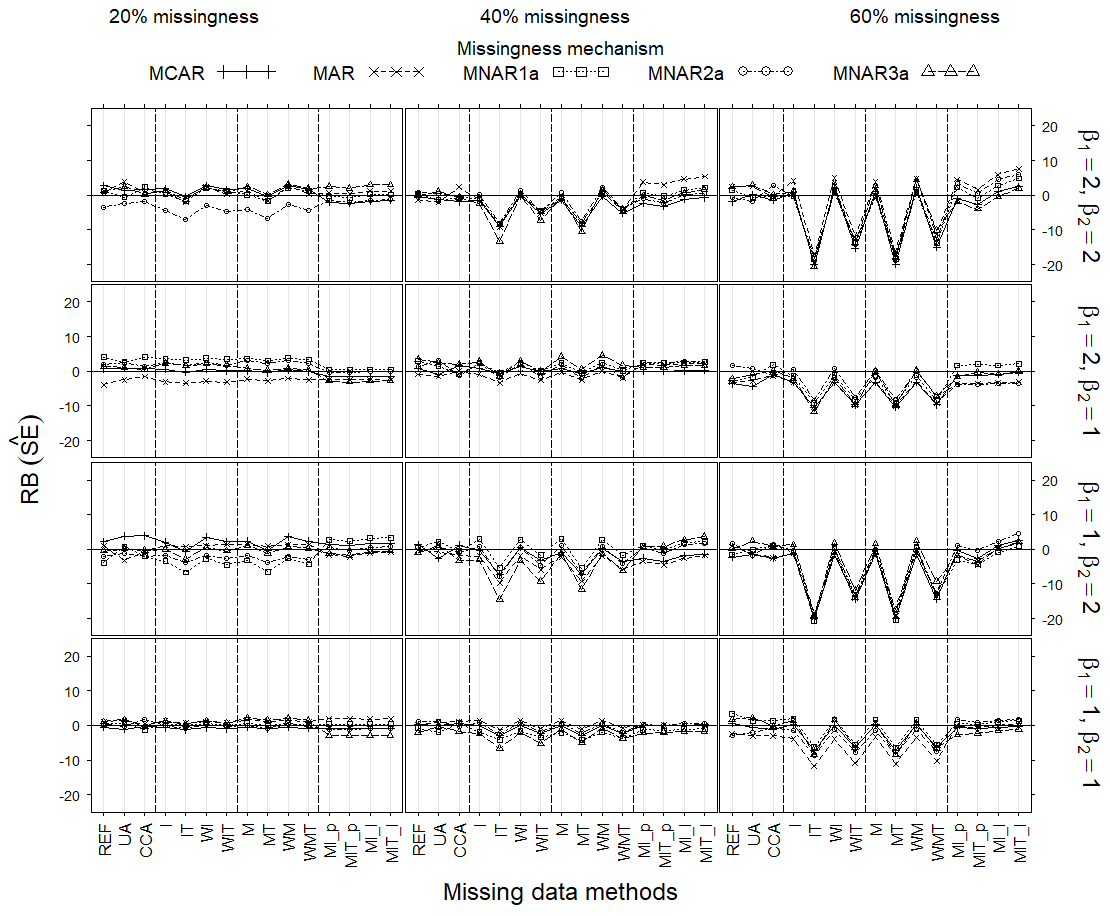


**Figure 9**. Relative bias (RB) of estimated standard error ($\hat{SE}$) (Y-axis) as a function of missingness method (X-axis), for each scenario as defined by missingness rate (left/middle/right columns), missingness mechanism (curves), true treatment effect (β_1_) and covariate effect (β_2_) (rows), and sample size 400. Note that (MCAR, MNAR1a) can always apply and (MAR, MNAR2a, MNAR3a) can only apply when the covariate is measured after randomization (but before treatment).


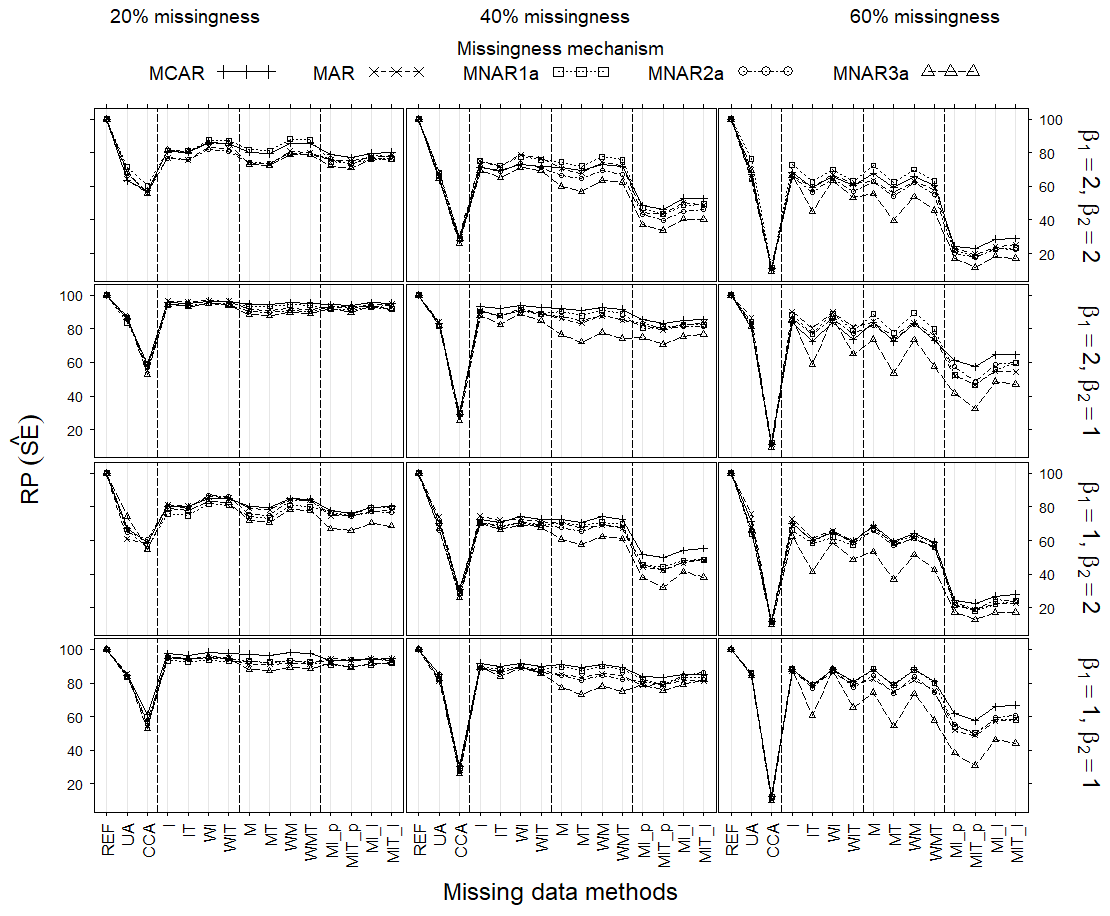


**Figure 10** Relative precision (RP) of estimated standard error ($\hat{SE}$) (Y-axis) as a function of missingness method (X-axis), for each scenario as defined by missingness rate (left/middle/right columns), missingness mechanism (curves), true treatment effect (β_1_) and covariate effect (β_2_) (rows), and sample size 400. Note that (MCAR, MNAR1a) can always apply and (MAR, MNAR2a, MNAR3a) can only apply when the covariate is measured after randomization (but before treatment).

Results under MCAR, MAR, MNAR1b, MNAR2b, and MNAR3b, for sample size 100


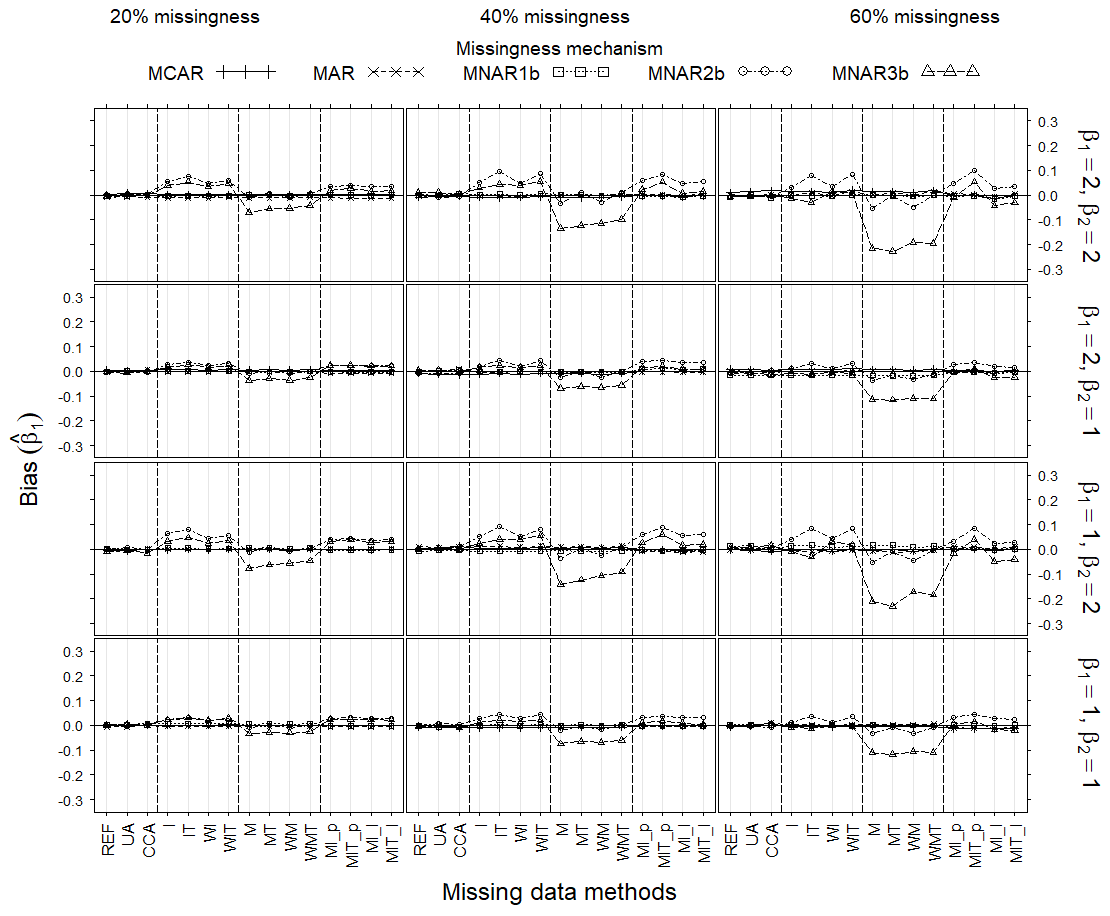


**Figure 11**. Bias of the treatment effect estimates ($Bias\left( \hat{\beta}_{1} \right)$) (Y-axis) as a function of missingness method (X-axis), for each scenario as defined by missingness rate (left/middle/right columns), missingness mechanism (curves), true treatment effect (β_1_) and covariate effect (β_2_) (rows), and sample size 100. Note that (MCAR, MNAR1b) can always apply and (MAR, MNAR2b, MNAR3b) can only apply when the covariate is measured after randomization (but before treatment).


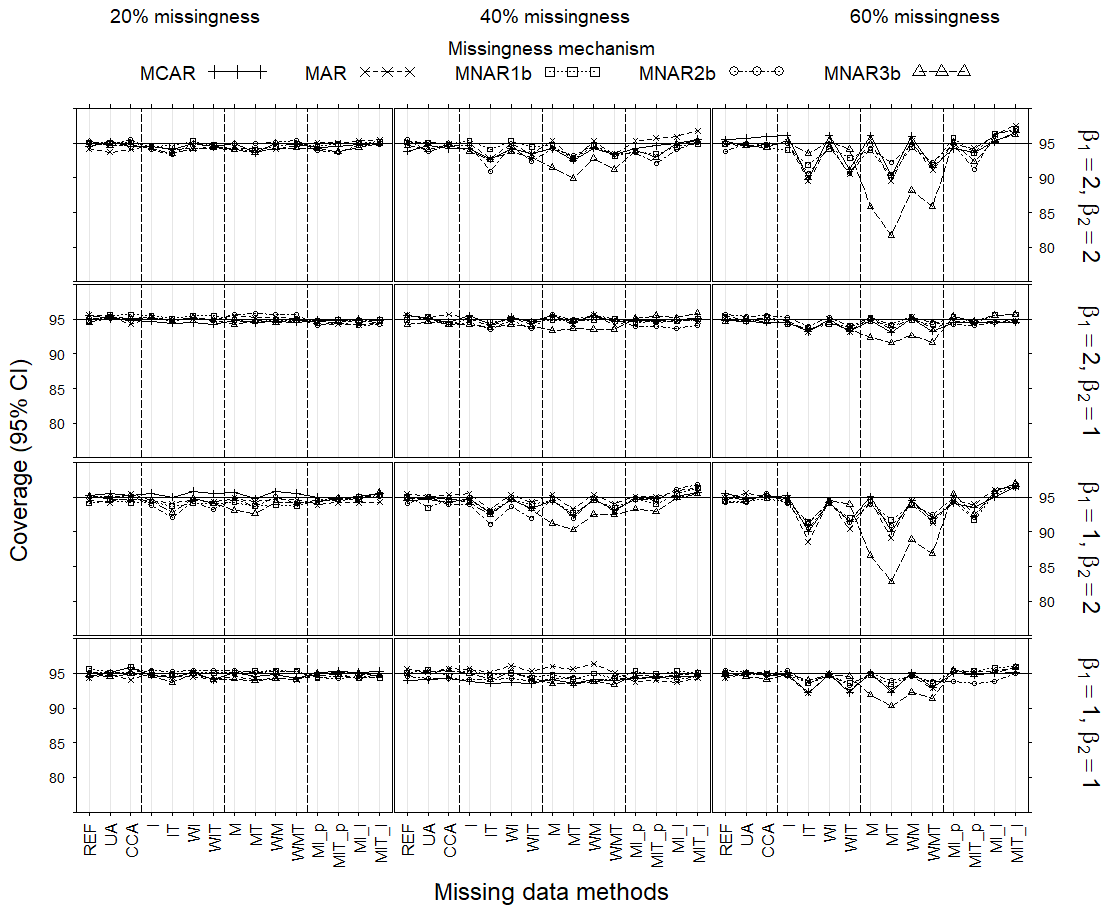


**Figure 12**. Coverage (%) of the 95% CI for the treatment effect estimate ($\hat{\beta}_{1}$) (Y-axis) as a function of missingness method (X-axis), for each scenario as defined by missingness rate (left/middle/right columns), missingness mechanism (curves), true treatment effect (β_1_) and covariate effect (β_2_) (rows), and sample size 100. Note that (MCAR, MNAR1b) can always apply and (MAR, MNAR2b, MNAR3b) can only apply when the covariate is measured after randomization (but before treatment).


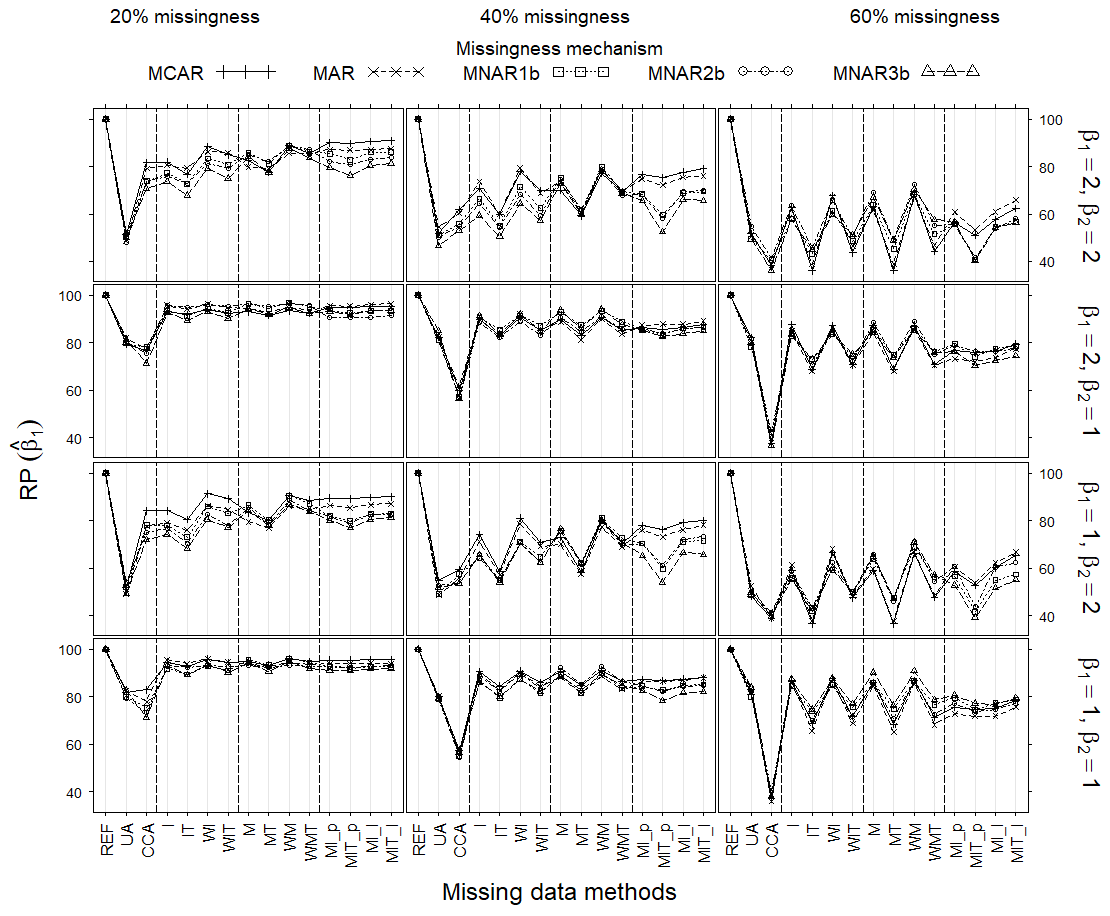


**Figure 13**. Relative precision (RP) of treatment effect estimate ($\hat{\beta}_{1}$) (Y-axis) as a function of missingness method (X-axis), for each scenario as defined by missingness rate (left/middle/right columns), missingness mechanism (curves), true treatment effect (β_1_) and covariate effect (β_2_) (rows), and sample size 100. Note that (MCAR, MNAR1b) can always apply and (MAR, MNAR2b, MNAR3b) can only apply when the covariate is measured after randomization (but before treatment).


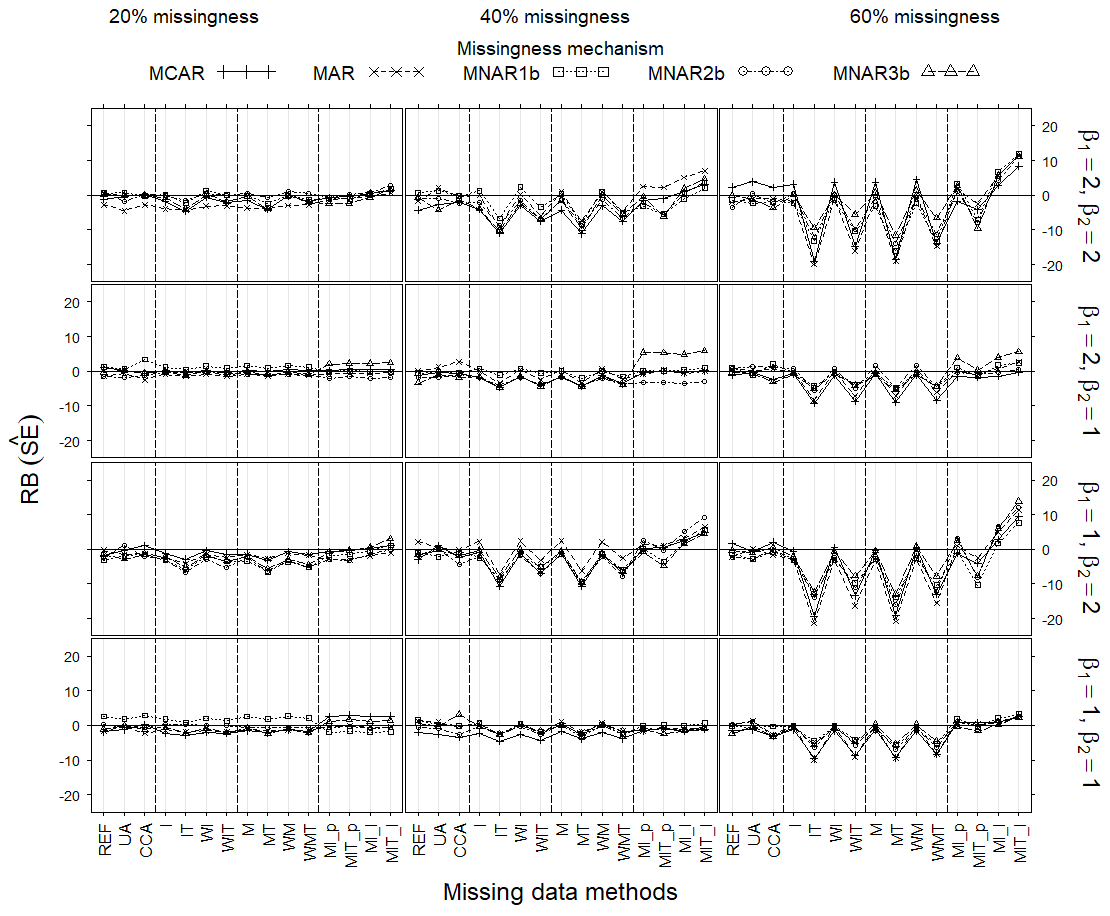


**Figure 14**. Relative bias (RB) of estimated standard error ($\hat{SE}$) (Y-axis) as a function of missingness method (X-axis), for each scenario as defined by missingness rate (left/middle/right columns), missingness mechanism (curves), true treatment effect (β_1_) and covariate effect (β_2_) (rows), and sample size 100. Note that (MCAR, MNAR1b) can always apply and (MAR, MNAR2b, MNAR3b) can only apply when the covariate is measured after randomization (but before treatment).


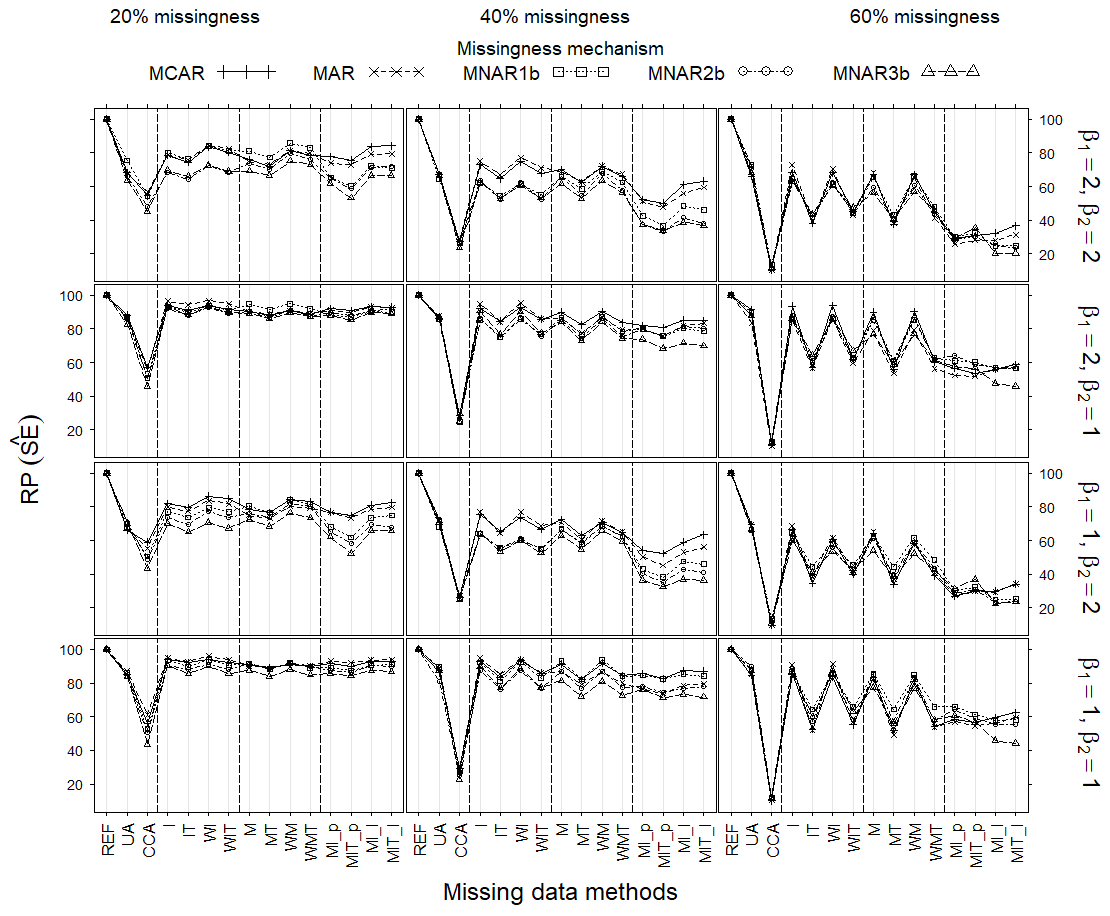


**Figure 15** Relative precision (RP) of estimated standard error ($\hat{SE}$) (Y-axis) as a function of missingness method (X-axis), for each scenario as defined by missingness rate (left/middle/right columns), missingness mechanism (curves), true treatment effect (β_1_) and covariate effect (β_2_) (rows), and sample size 100. Note that (MCAR, MNAR1b) can always apply and (MAR, MNAR2b, MNAR3b) can only apply when the covariate is measured after randomization (but before treatment).

Results under MCAR, MAR, MNAR1b, MNAR2b, and MNAR3b, for sample size 400


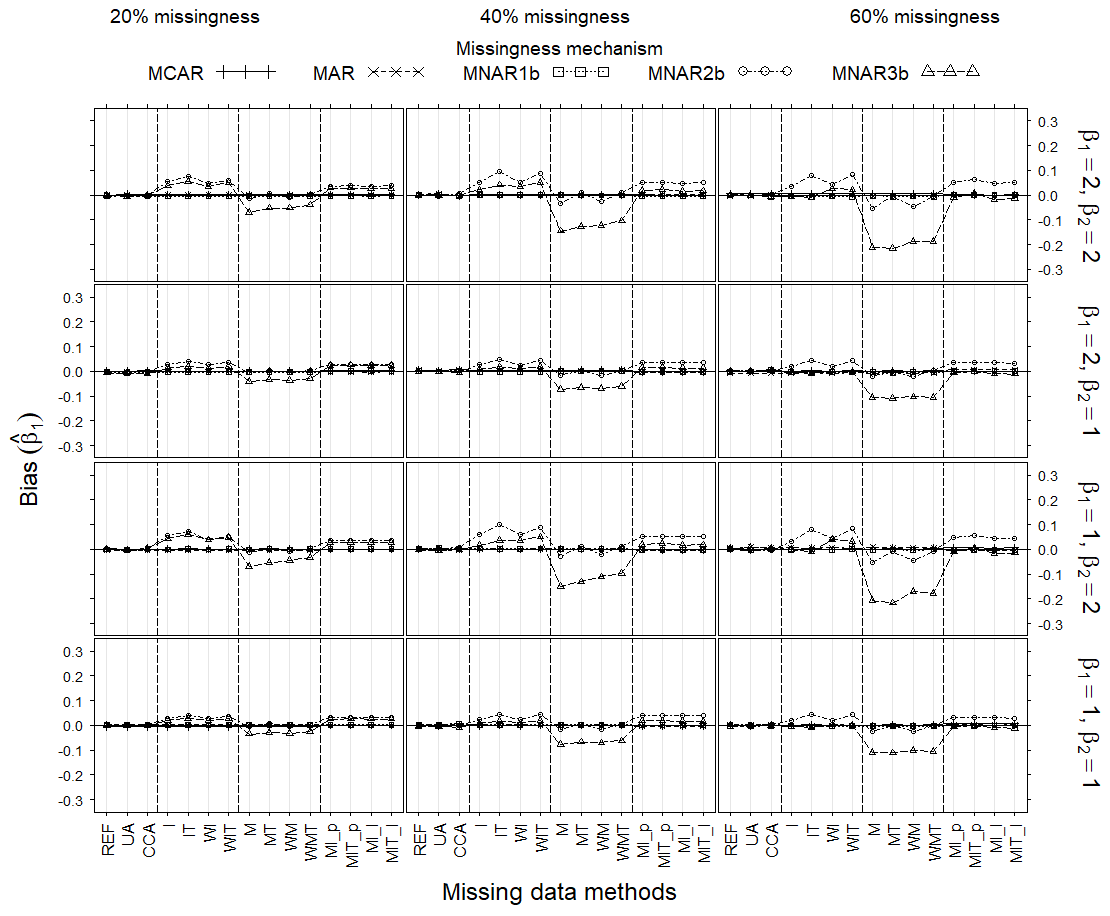


**Figure 16**. Bias of the treatment effect estimates ($Bias\left( \hat{\beta}_{1} \right)$) (Y-axis) as a function of missingness method (X-axis), for each scenario as defined by missingness rate (left/middle/right columns), missingness mechanism (curves), true treatment effect (β_1_) and covariate effect (β_2_) (rows), and sample size 400. Note that (MCAR, MNAR1b) can always apply and (MAR, MNAR2b, MNAR3b) can only apply when the covariate is measured after randomization (but before treatment).


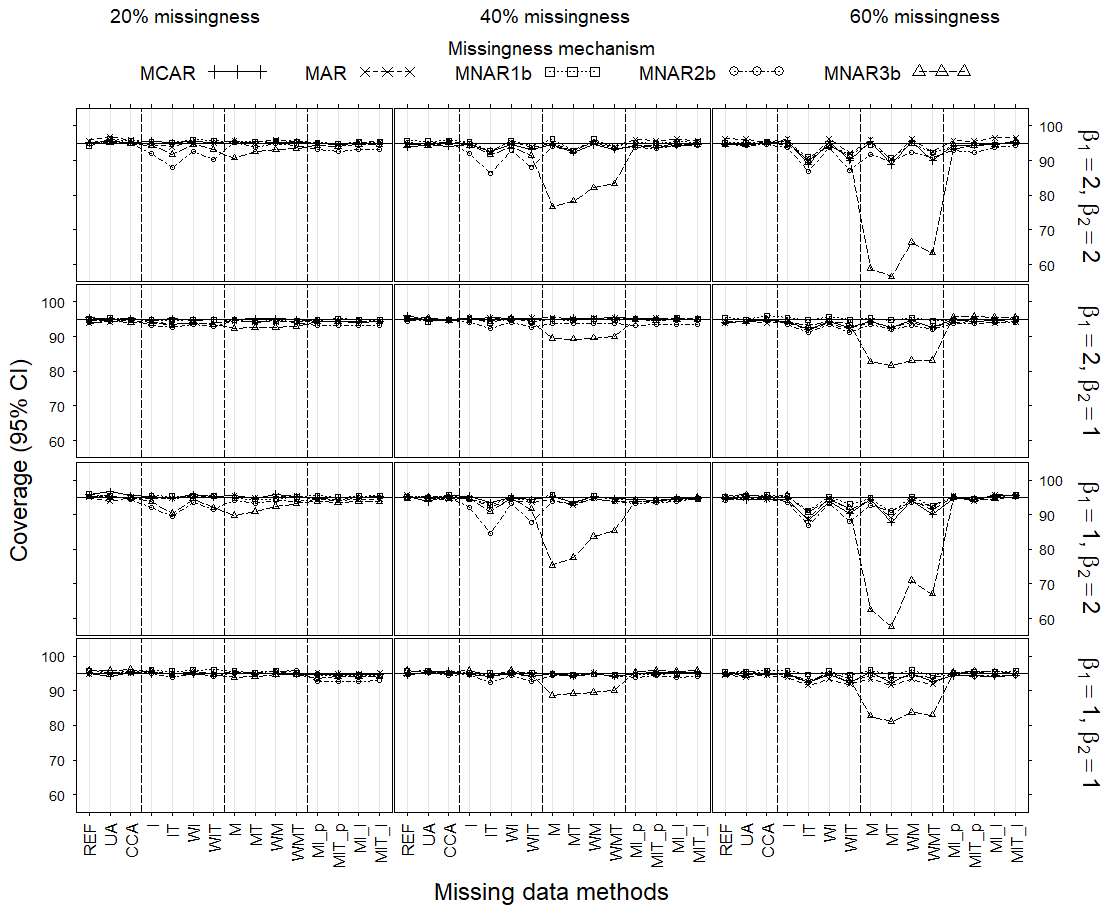


**Figure 17**. Coverage (%) of the 95% CI for the treatment effect estimate ($\hat{\beta}_{1}$) (Y-axis) as a function of missingness method (X-axis), for each scenario as defined by missingness rate (left/middle/right columns), missingness mechanism (curves), true treatment effect (β_1_) and covariate effect (β_2_) (rows), and sample size 400. Note that (MCAR, MNAR1b) can always apply and (MAR, MNAR2b, MNAR3b) can only apply when the covariate is measured after randomization (but before treatment).


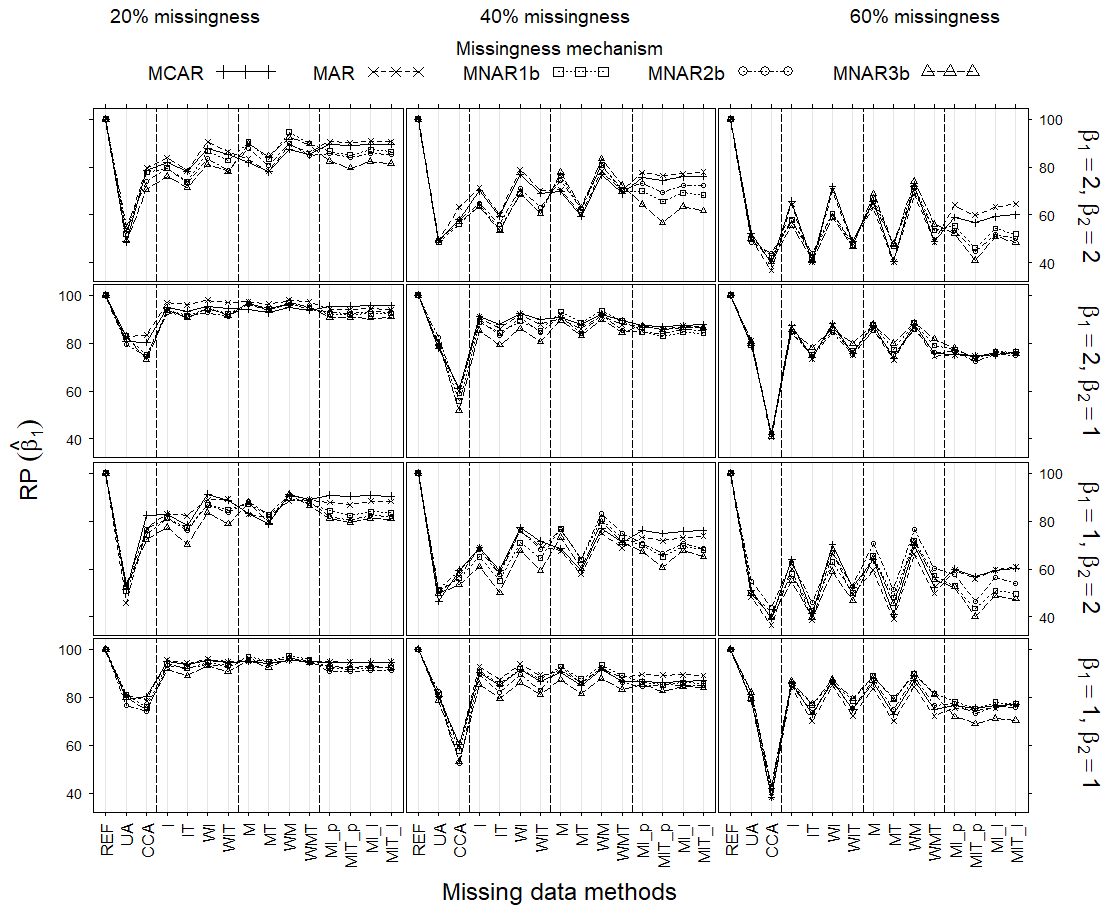


**Figure 18**. Relative precision (RP) of treatment effect estimate ($\hat{\beta}_{1}$) (Y-axis) as a function of missingness method (X-axis), for each scenario as defined by missingness rate (left/middle/right columns), missingness mechanism (curves), true treatment effect (β_1_) and covariate effect (β_2_) (rows), and sample size 400. Note that (MCAR, MNAR1b) can always apply and (MAR, MNAR2b, MNAR3b) can only apply when the covariate is measured after randomization (but before treatment).


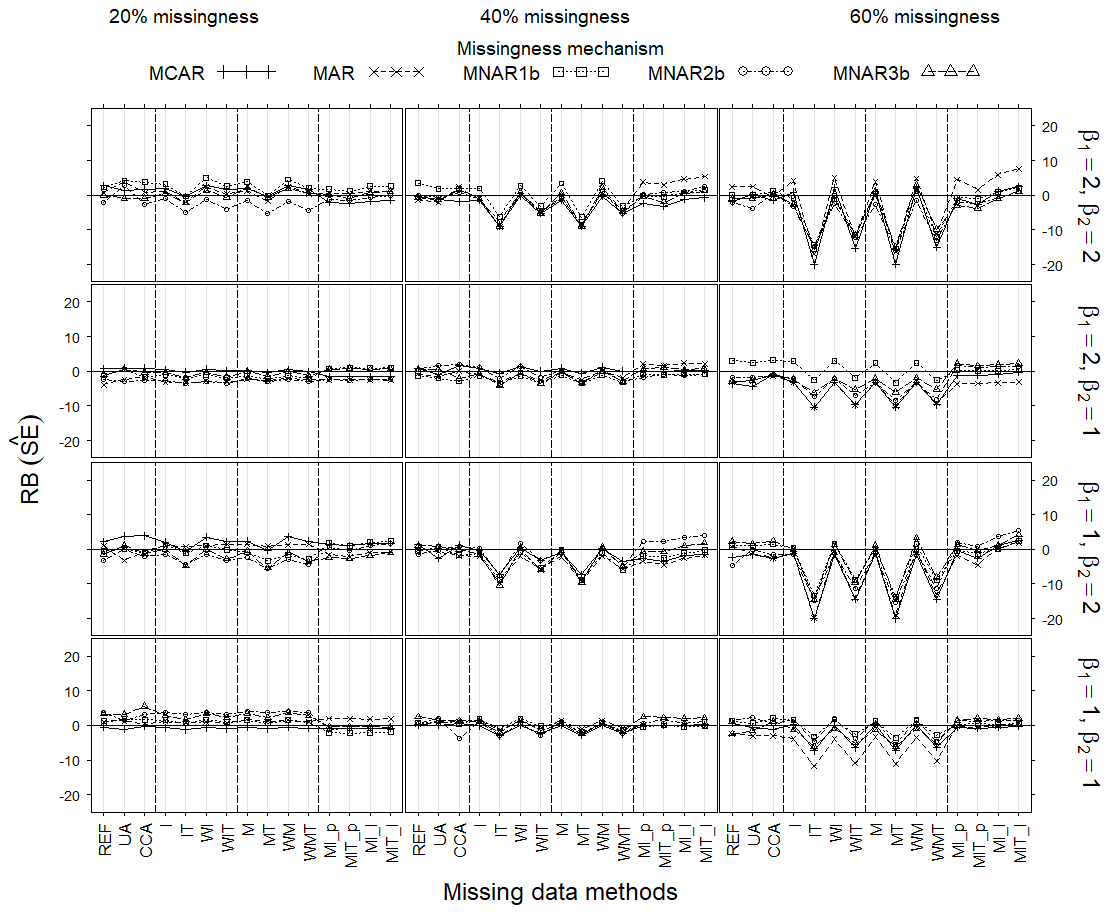


**Figure 19**. Relative bias (RB) of estimated standard error ($\hat{SE}$) (Y-axis) as a function of missingness method (X-axis), for each scenario as defined by missingness rate (left/middle/right columns), missingness mechanism (curves), true treatment effect (β_1_) and covariate effect (β_2_) (rows), and sample size 400. Note that (MCAR, MNAR1b) can always apply and (MAR, MNAR2b, MNAR3b) can only apply when the covariate is measured after randomization (but before treatment).


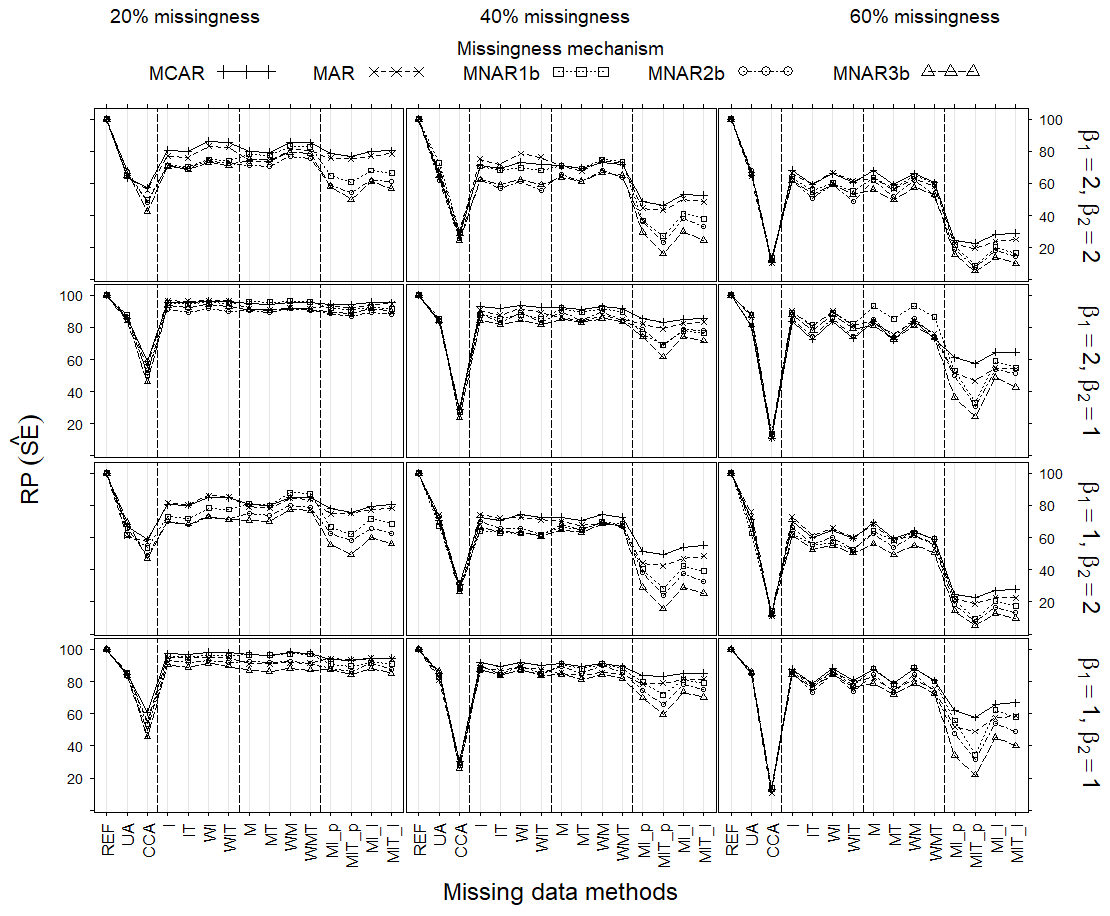


**Figure 20** Relative precision (RP) of estimated standard error ($\hat{SE}$) (Y-axis) as a function of missingness method (X-axis), for each scenario as defined by missingness rate (left/middle/right columns), missingness mechanism (curves), true treatment effect (β_1_) and covariate effect (β_2_) (rows), and sample size 400. Note that (MCAR, MNAR1b) can always apply and (MAR, MNAR2b, MNAR3b) can only apply when the covariate is measured after randomization (but before treatment).

Extension to time-to-event outcome

### **Results under a-scenarios Table 3, with sample size 100**


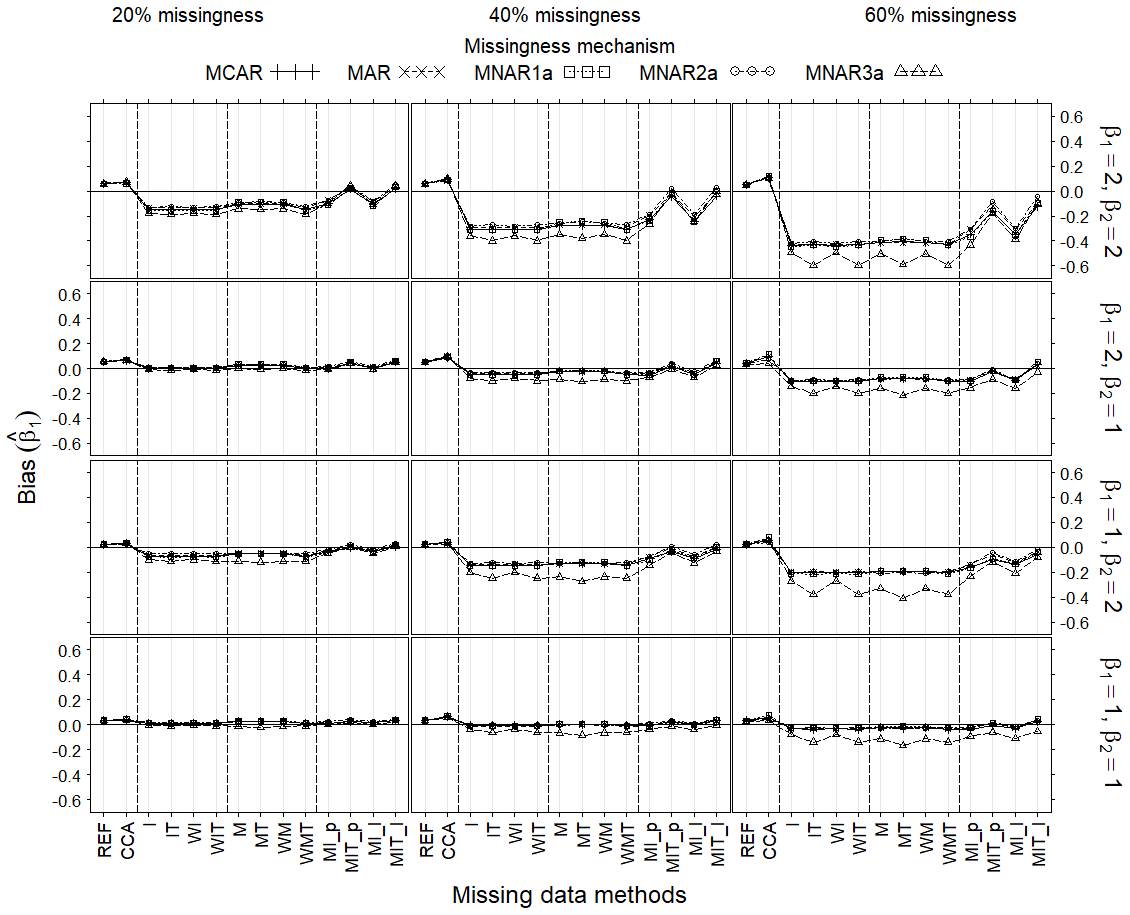


**Figure 21**. Bias of the treatment effect estimates ($Bias\left( \hat{\beta}_{1} \right)$) (Y-axis) as a function of missingness method (X-axis), for each scenario as defined by missingness rate (left/middle/right columns), missingness mechanism (curves), true treatment effect (β_1_) and covariate effect (β_2_) (rows), and sample size 100. Note that (MCAR, MNAR1a) can always apply and (MAR, MNAR2a, MNAR3a) can only apply when the covariate is measured after randomization (but before treatment).


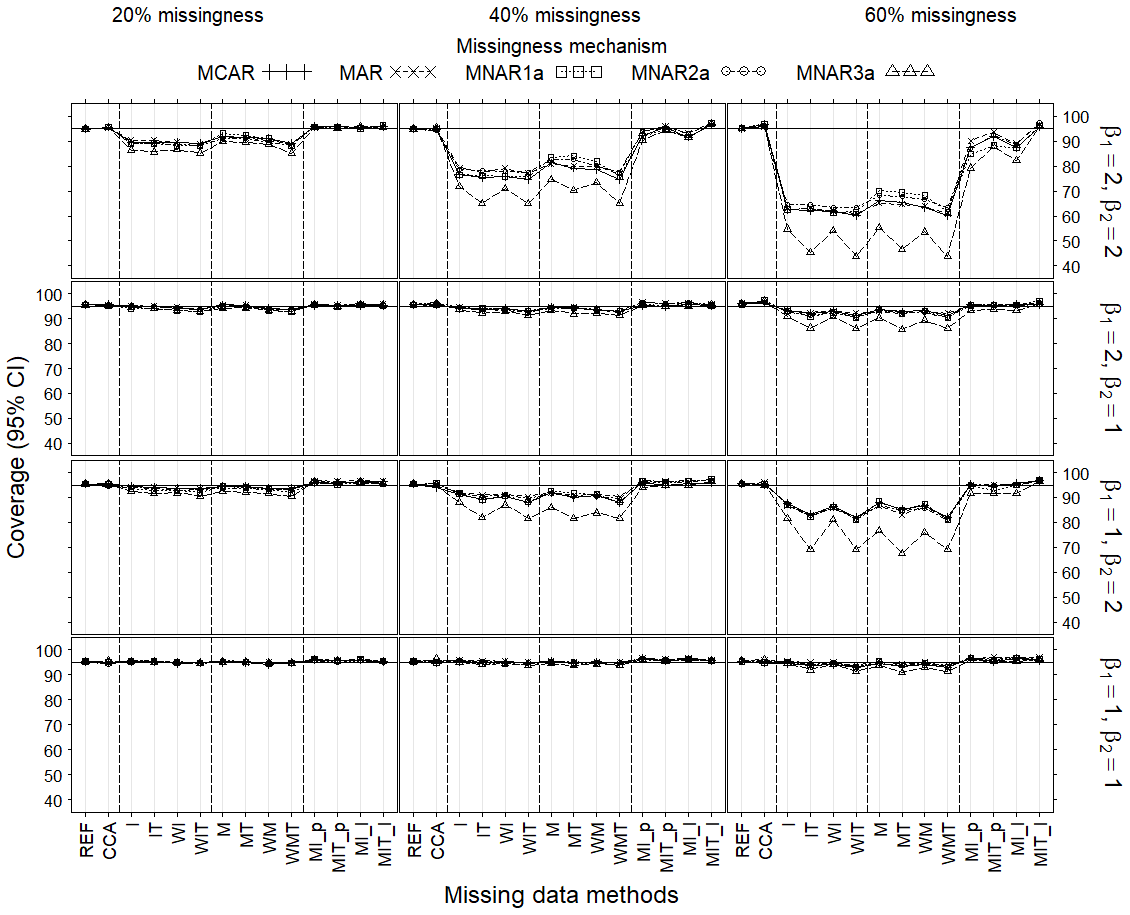


**Figure 22**. Coverage (%) of the 95% CI for the treatment effect estimate ($\hat{\beta}_{1}$) (Y-axis) as a function of missingness method (X-axis), for each scenario as defined by missingness rate (left/middle/right columns), missingness mechanism (curves), true treatment effect (β_1_) and covariate effect (β_2_) (rows), and sample size 100. Note that (MCAR, MNAR1a) can always apply and (MAR, MNAR2a, MNAR3a) can only apply when the covariate is measured after randomization (but before treatment).


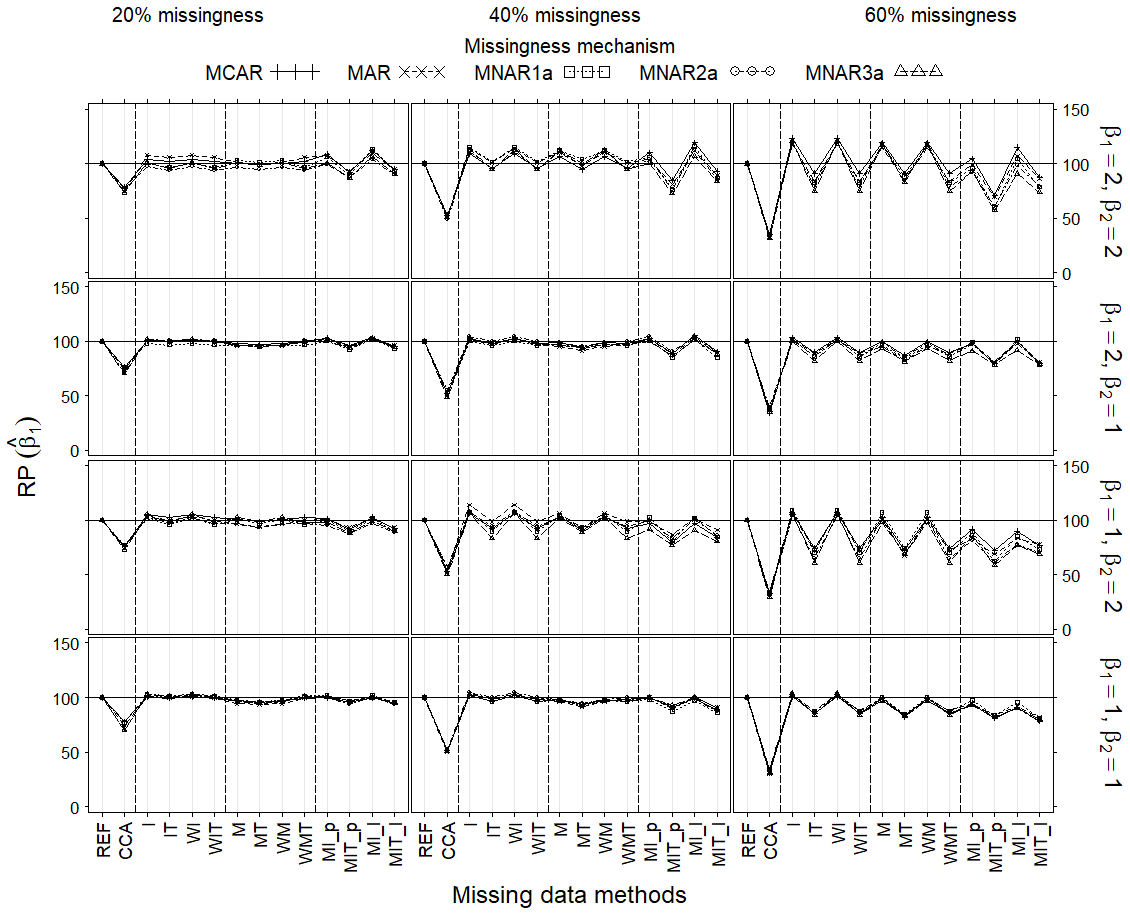


**Figure 23**. Relative precision (RP) of treatment effect estimate ($\hat{\beta}_{1}$) (Y-axis) as a function of missingness method (X-axis), for each scenario as defined by missingness rate (left/middle/right columns), missingness mechanism (curves), true treatment effect (β_1_) and covariate effect (β_2_) (rows), and sample size 100. Note that (MCAR, MNAR1a) can always apply and (MAR, MNAR2a, MNAR3a) can only apply when the covariate is measured after randomization (but before treatment).


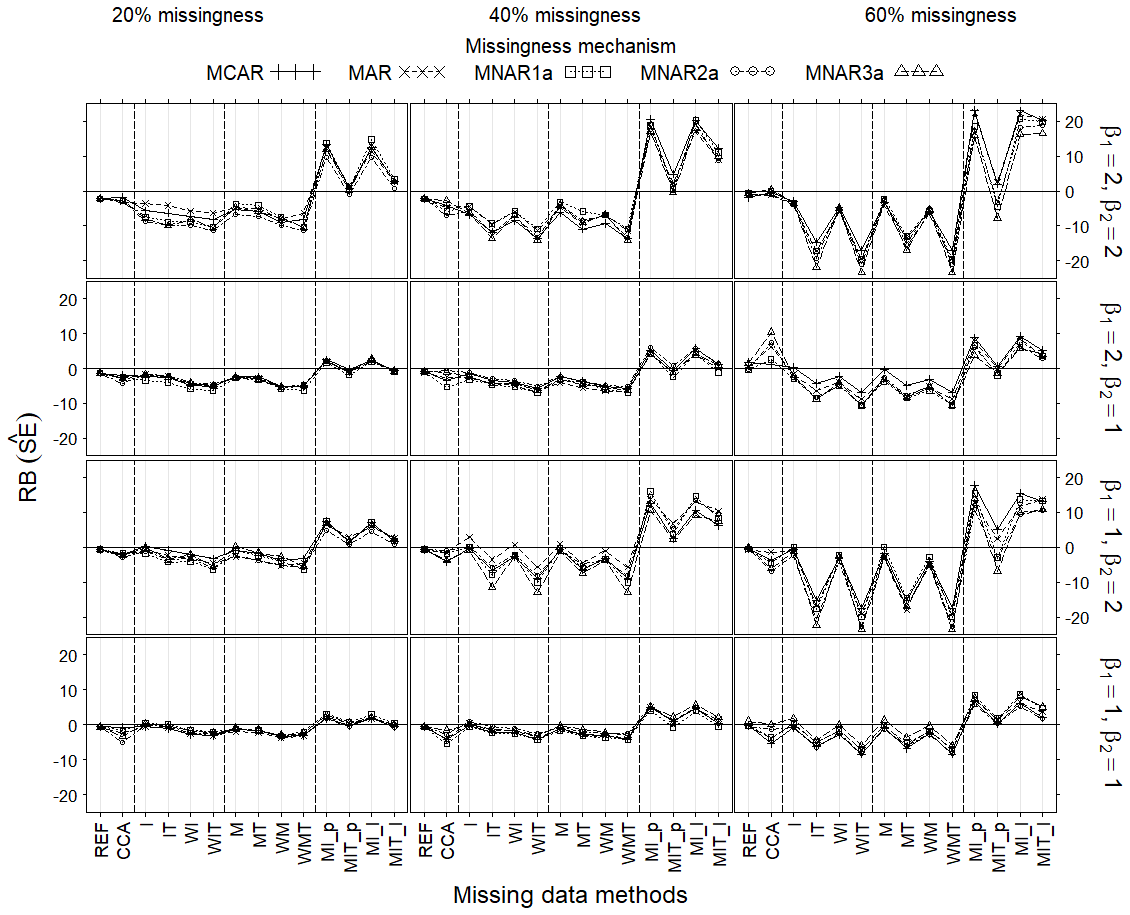


**Figure 24**. Relative bias (RB) of estimated standard error ($\hat{SE}$) (Y-axis) as a function of missingness method (X-axis), for each scenario as defined by missingness rate (left/middle/right columns), missingness mechanism (curves), true treatment effect (β_1_) and covariate effect (β_2_) (rows), and sample size 100. Note that (MCAR, MNAR1a) can always apply and (MAR, MNAR2a, MNAR3a) can only apply when the covariate is measured after randomization (but before treatment).


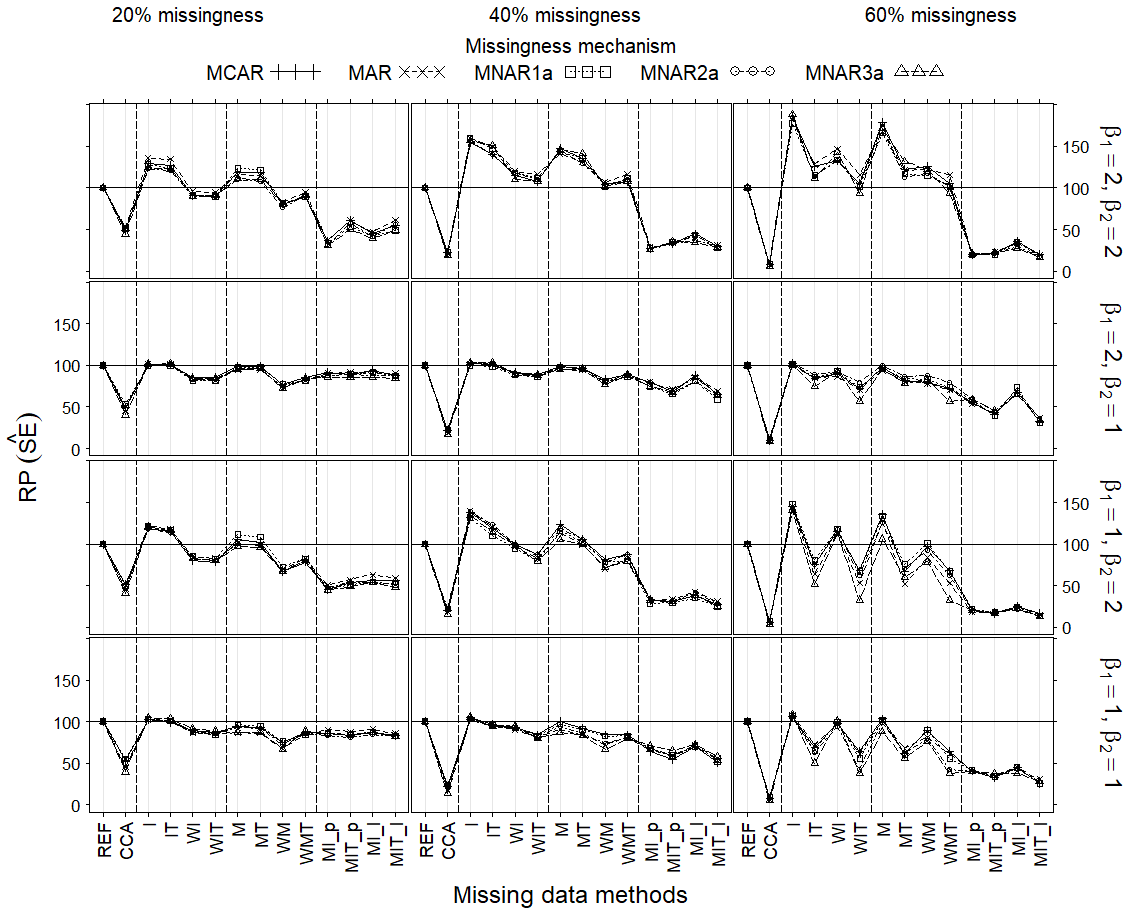


**Figure 25** Relative precision (RP) of estimated standard error ($\hat{SE}$) (Y-axis) as a function of missingness method (X-axis), for each scenario as defined by missingness rate (left/middle/right columns), missingness mechanism (curves), true treatment effect (β_1_) and covariate effect (β_2_) (rows), and sample size 100. Note that (MCAR, MNAR1a) can always apply and (MAR, MNAR2a, MNAR3a) can only apply when the covariate is measured after randomization (but before treatment).

Results under b-scenarios in Table 3, with sample size 100


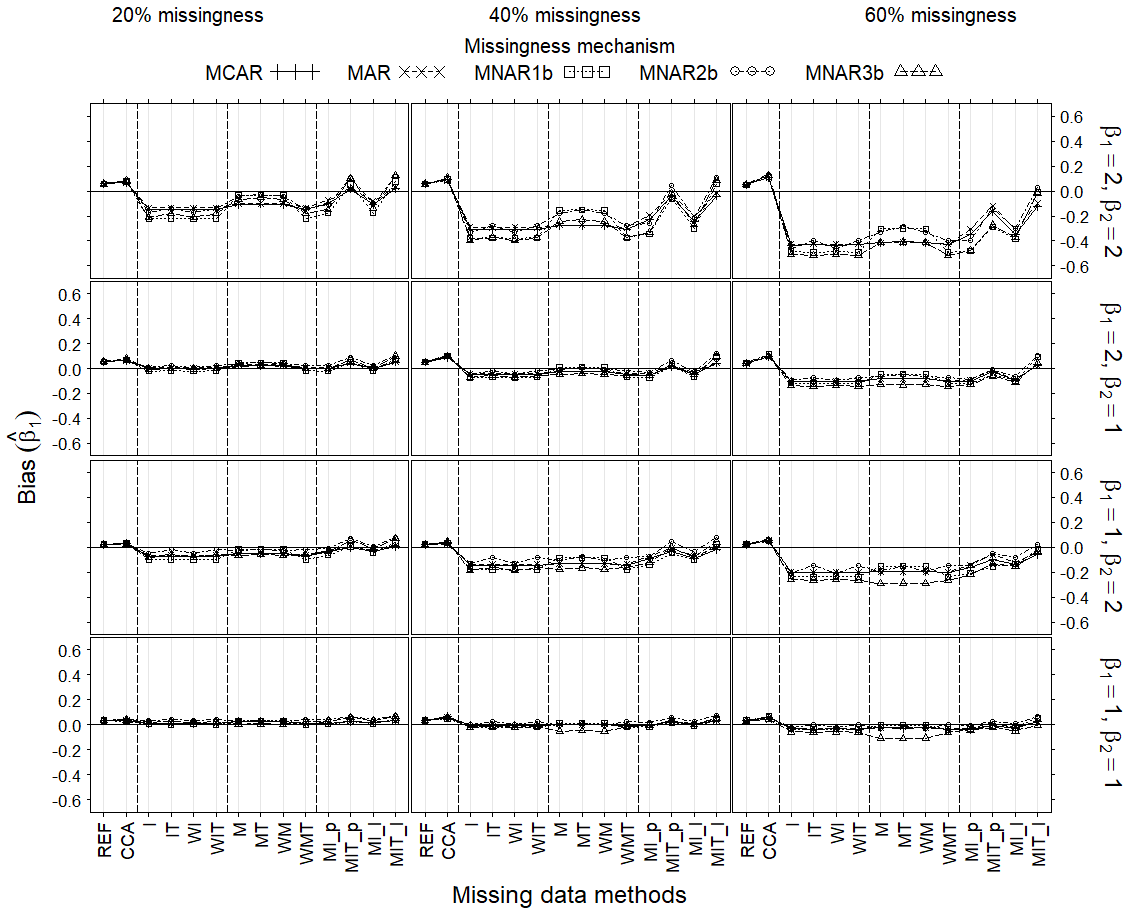


**Figure 26**. Bias of the treatment effect estimates ($Bias\left( \hat{\beta}_{1} \right)$) (Y-axis) as a function of missingness method (X-axis), for each scenario as defined by missingness rate (left/middle/right columns), missingness mechanism (curves), true treatment effect (β_1_) and covariate effect (β_2_) (rows), and sample size 100. Note that (MCAR, MNAR1b) can always apply and (MAR, MNAR2b, MNAR3b) can only apply when the covariate is measured after randomization (but before treatment).


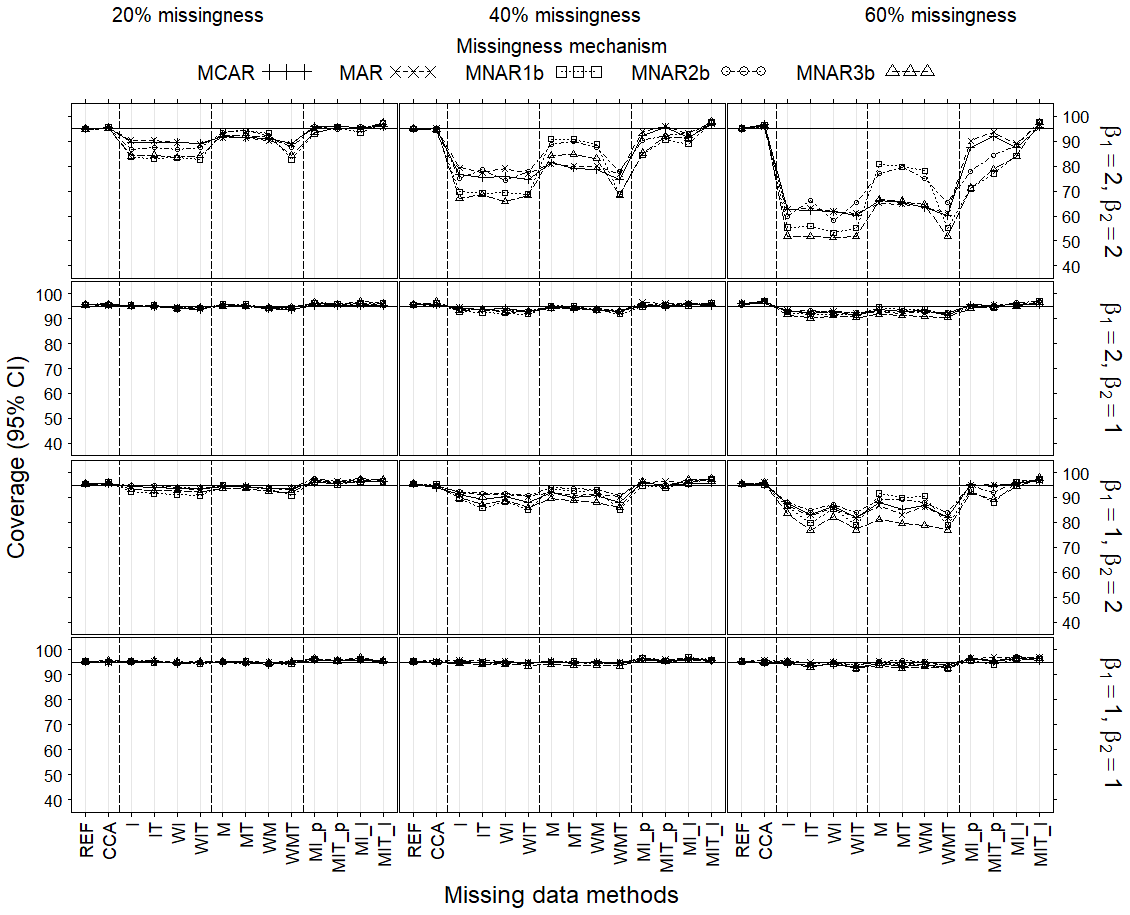


**Figure 27**. Coverage (%) of the 95% CI for the treatment effect estimate ($\hat{\beta}_{1}$) (Y-axis) as a function of missingness method (X-axis), for each scenario as defined by missingness rate (left/middle/right columns), missingness mechanism (curves), true treatment effect (β_1_) and covariate effect (β_2_) (rows), and sample size 100. Note (MCAR, MNAR1b) can always apply and (MAR, MNAR2b, MNAR3b) can only apply when the covariate is measured after randomization (but before treatment).


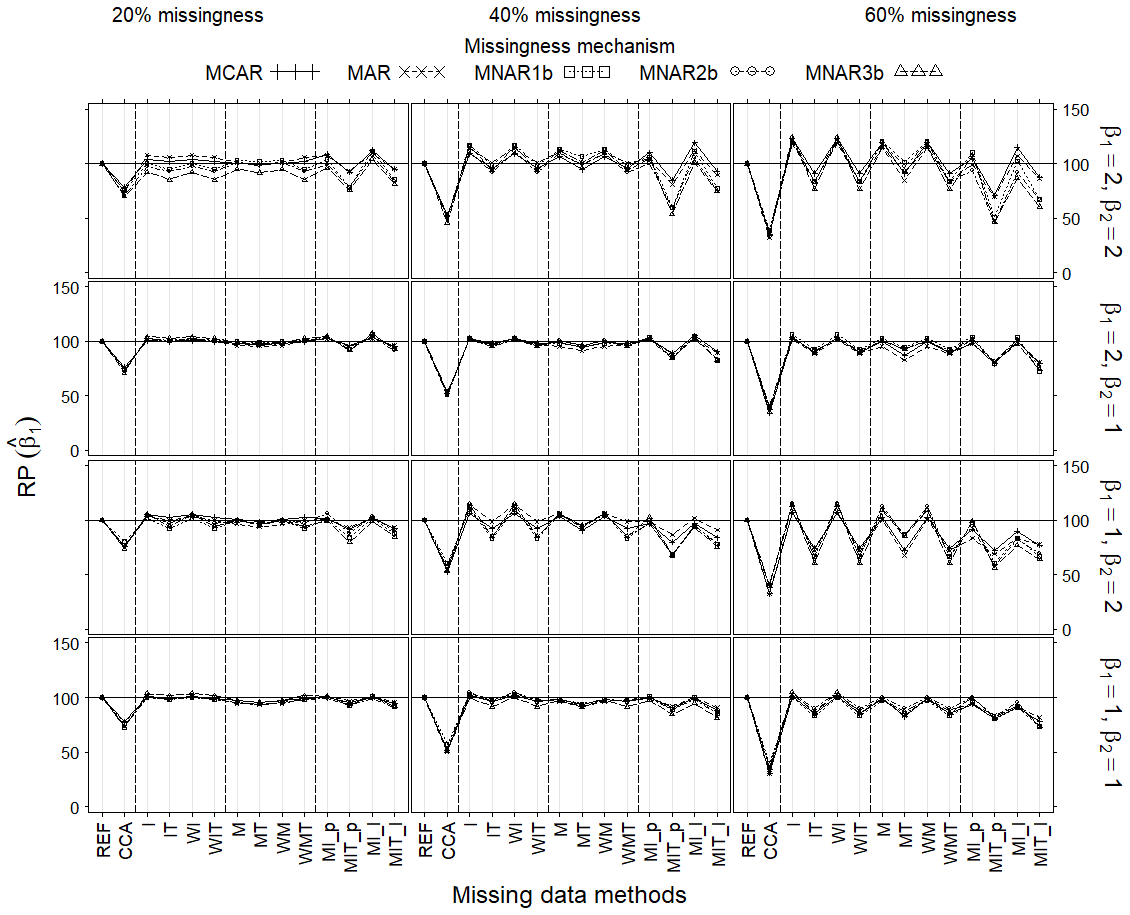


**Figure 28**. Relative precision (RP) of treatment effect estimate ($\hat{\beta}_{1}$) (Y-axis) as a function of missingness method (X-axis), for each scenario as defined by missingness rate (left/middle/right columns), missingness mechanism (curves), true treatment effect (β_1_) and covariate effect (β_2_) (rows), and sample size 100. Note that (MCAR, MNAR1b) can always apply and (MAR, MNAR2b, MNAR3b) can only apply when the covariate is measured after randomization (but before treatment).


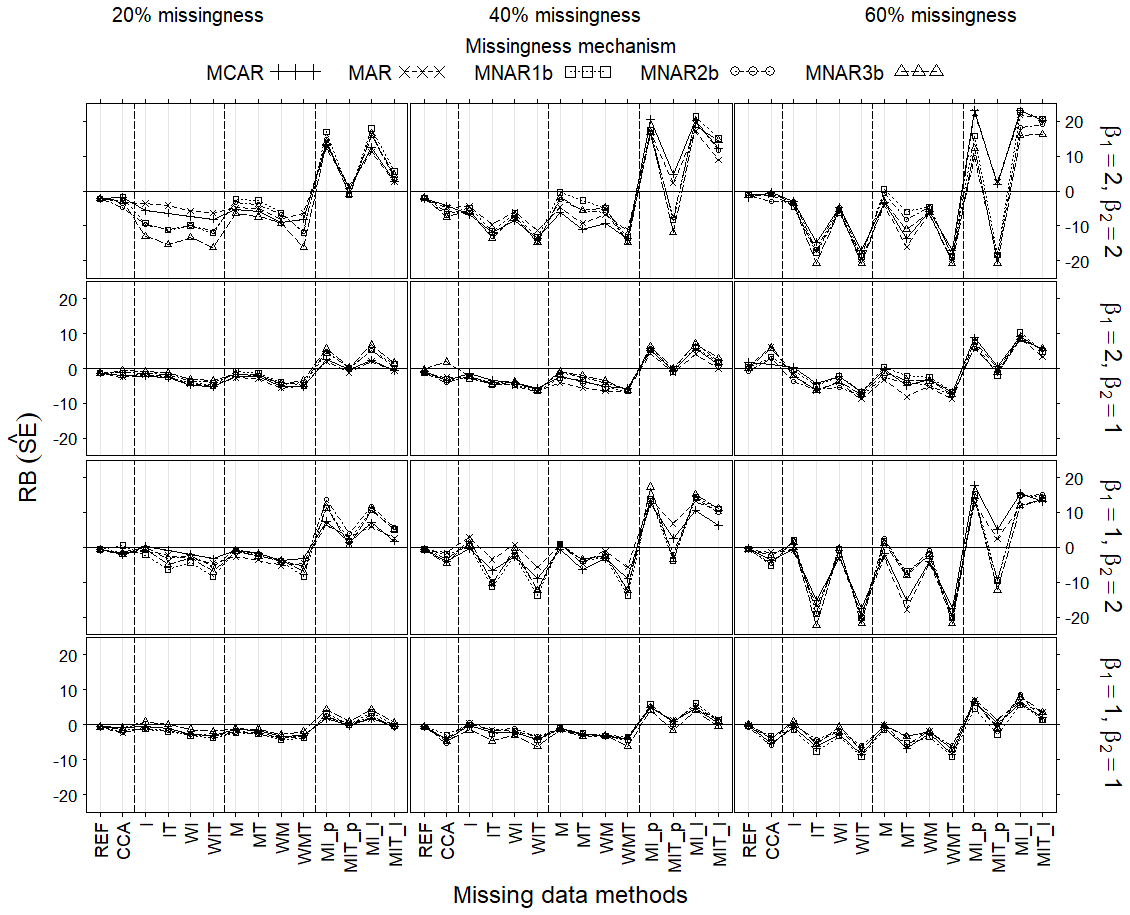


**Figure 29**. Relative bias (RB) of estimated standard error ($\hat{SE}$) (Y-axis) as a function of missingness method (X-axis), for each scenario as defined by missingness rate (left/middle/right columns), missingness mechanism (curves), true treatment effect (β_1_) and covariate effect (β_2_) (rows), and sample size 100. Note that (MCAR, MNAR1b) can always apply and (MAR, MNAR2b, MNAR3b) can only apply when the covariate is measured after randomization (but before treatment).


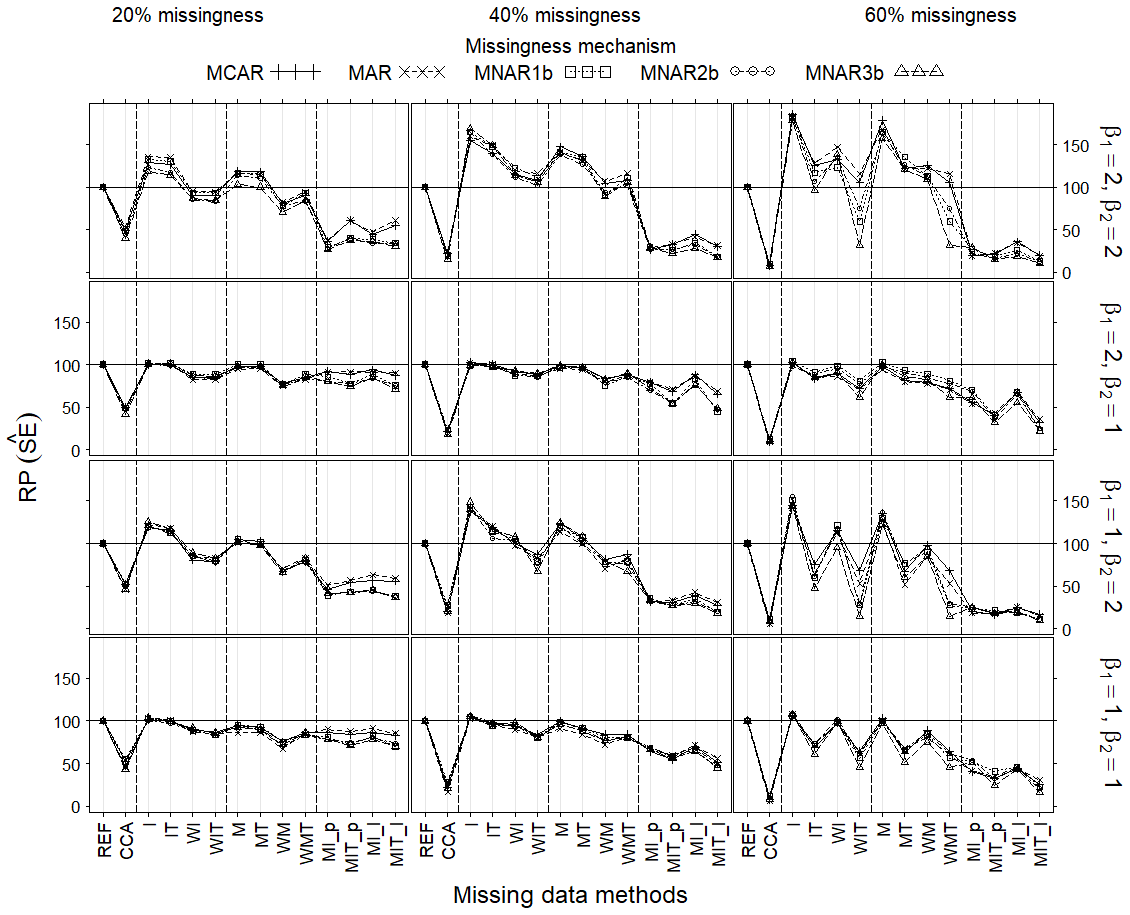


**Figure 30** Relative precision (RP) of estimated standard error ($\hat{SE}$) (Y-axis) as a function of missingness method (X-axis), for each scenario as defined by missingness rate (left/middle/right columns), missingness mechanism (curves), true treatment effect (β_1_) and covariate effect (β_2_) (rows), and sample size 100. Note that (MCAR, MNAR1b) can always apply and (MAR, MNAR2b, MNAR3b) can only apply when the covariate is measured after randomization (but before treatment).
